# A multiplex metabolomic approach for quality control of Spirulina supplement and its allied microalgae (Amphora & Chlorella) assisted by chemometrics and molecular networking

**DOI:** 10.1038/s41598-024-53219-5

**Published:** 2024-02-02

**Authors:** Nesrine Hegazi, Amira R. Khattab, Hamada H. Saad, Bishoy Abib, Mohamed A. Farag

**Affiliations:** 1 Department of Phytochemistry and Plant Systematics, National Research Centre, Dokki, 12622 Cairo Egypt; 2 Pharmacognosy Department, College of Pharmacy, Arab Academy for Science, Technology and Maritime Transport, Alexandria, 1029 Egypt; 3 Chemistry Department, American University in Cairo, Cairo, Egypt; 4 Pharmacognosy Department, College of Pharmacy, Cairo University, Kasr El Aini St., P.B. 11562, Cairo, Egypt

**Keywords:** Plant sciences, Chemistry

## Abstract

Microalgae species are of economic importance regarded as “green gold” being rich in bioactive compounds. *Spirulina* and *Chlorella* are the most popular microalgal species and are marketed as healthy food supplements. At the same time, *Amphora* holds potential as a source of healthy lipids and essential fatty acids. Yet, there are considerable variations in their reported chemical composition, and less is known about their compositional differences. A multiplexed metabolomic approach was adopted for the quality control (QC) of *Spirulina* supplements and to compare its constitutive metabolome to *Chlorella* and *Amphora*. The adopted protocol comprised gas chromatography-mass spectrometry (GC–MS), ultra-high performance liquid chromatography coupled with high-resolution tandem mass spectrometry (UPLC-HRMS/MS), and ultraviolet–visible spectrophotometry (UV/Vis) for mapping their primary and secondary metabolome. Interestingly, UPLC-HRMS/MS analysis delineated the abundance of fatty acids in *Amphora versus* glycolipids enrichment in *Spirulina*, and porphyrins were the main pigments identified in *Spirulina*, with scarce occurrence in *Chlorella*. Orthogonal projections to latent structures discriminant analysis (OPLS-DA) analysis of GC–MS data set revealed palmitic acid, 3-mannobiose, and glyceryl-glycoside as being most enriched in *Spirulina*, versus sucrose and leucine in *Chlorella* and *Amphora*, respectively. Despite being of low discriminatory potential, UV/Vis OPLS-DA modeling showed that *Spirulina* was distinguished with the UV absorbances of carotenoids and chlorophyll pigments, as indicated by its OPLS-DA derived S-plot. Our study provides a QC approach for the analysis of the microalgal species and poses alternative spectral and compositional markers for their discrimination.

## Introduction

Nowadays, sustainable food production is a pressing need to combat the scarcity of natural resources and reduce associated environmental effects^1^. Transformation to sustainable food production aims at providing a globally accessible healthier diet. Healthier diets should include food rich in polyunsaturated fatty acids (i.e., omega-3 and omega-6 fatty acids). Commonly fish oil is the most consumed for its omega-3 content, yet there is a growing necessity to find an alternative source for the limits of fishing and to meet the needs of vegetarians and vegans^2^. To fill such a need, microalgae have attracted increasing attention as a rich source of omega-3 and omega-6 lipids. Besides being nutritional rich, they are grown under sustainable conditions, thus helping us preserve our scarce ocean resources^3^. Microalage compromises a massive number of species ranging from 200,000 to 800,000^4^. Some species are of economic and industrial importance and could be regarded as “green gold” being a source of bioactive compounds for the production of food, feed, cosmetics, and biofuel^5^. Besides being abundant in proteins, carbohydrates, and lipids, they are also a superior resource of vitamins and minerals^6^. Compared to extensive phytochemical studies on plant-based nutraceuticals^7^, much less is reported in the literature regarding marine-based neutraceuticals, including those derived from microalgae and their commercial preparations.

Presently, microalgae are promoted as healthy food and are available as dietary supplements in different forms i.e. powders, tablets, capsules, and liquids^8^. Among the widely consumed microalgae species are *Spirulina* (*Arthrospira maxima* and *Arythrospira platensis*) and *Chlorella* sp., which are dominating the global market due to their nutrient-rich profile^9^. In addition, they are also used as feed for terrestrial and aquatic animals^10^. According to WHO, *Spirulina* is recognized as a “superfood” and is marketed by NASA as the most concentrated human food^11^. Many studies have dealt with its chemical composition. *Chlorella sp.* as well is industrially cultivated as human food and is recognized for its high lipid content (specially enriched in omega-3 and omega-6 fatty acids)^3^. Yet, there are considerable variations in the reported chemical composition of the same algal species or even strains which could be attributed to differences in the cultivation conditions^12^.

Besides the widely recognized *Spirulina* and *Chlorella*, *Amphora* sp. holds potential as an alternative source of healthy lipids and essential fatty acids. *Amphora* is a pennate diatom microalga, which recently has attracted interest as a biofuel, and as a source of essential lipids being highly productive and environmentally friendly^12^.

**Figure 1:**
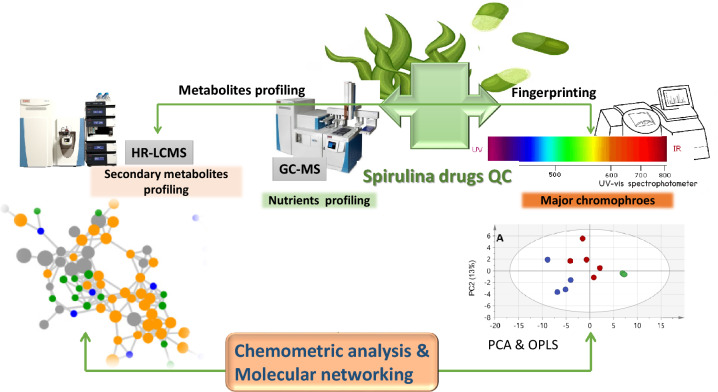
Schematic representation of the adopted metabolomics workflow for the analysis of *Spirulina* supplements in comparison to other microalgal species (i.e.* Chlorella* and *Amphora*).

In this regard, a multiplex metabolome approach was undertaken to map the metabolome of different commercially marketed *Spirulina* samples and to compare it to those of *Chlorella* and *Amphora* species as other microalgae. Metabolomics provides a comprehensive insight into organisms' chemical composition, increasingly reported for quality control of herbal drugs and less reported in the case of marine nutraceuticals. Metabolomics typically employs chromatographic tools, i.e., gas chromatography (GC) or liquid chromatography (LC), coupled to spectroscopic tools, i.e., MS or NMR, for metabolites detection, followed by analysis using multivariate data analyses for data visualization. In this study, metabolomics tools, including ultra-high performance liquid chromatography coupled to high-resolution tandem mass spectrometry *(*UPLC-HRMS/MS) and gas chromatography coupled to mass spectrometry (GC–MS) post-silylation were recruited for such a purpose. Recently, metabolomics has been an irreplaceable approach for fingerprinting and analyzing complex food matrices, offering valuable insights into their composition and their nutritive value^13^, and is scarcely applied in algae analysis. Additionally, their discrimination based on their UV–vis spectroscopic profile was compared to their acquired metabolomics data considering its robustness, less expensive, and more applicable at the industrial scale^14^. Owing to the complexity of metabolomics-driven data, multivariate data analysis was recruited to further determine discriminating metabolites among algal samples and identify markers for each taxon. Additionally, an inspection of the recorded metabolome (i.e., UPLC-HRMS/MS data) was aided by the spectral similarities network available through the GNPS platform (https://gnps.ucsd.edu) for the first time to be reported in algae, which allows for propagation of the metabolites annotation and mapping metabolites distribution among the studied samples (Fig. 1).

## Results and discussion

### UPLC-HRMS/MS analysis of *Spirulina* samples versus *Amphora* and *Chlorella*

The current study aimed to broadly compare the metabolome of *Spirulina* samples in context to those of other nutritionally significant algal species (i.e.* Chlorella* and *Amphora*) targeting their large secondary and primary metabolome.

**Figure 2:**
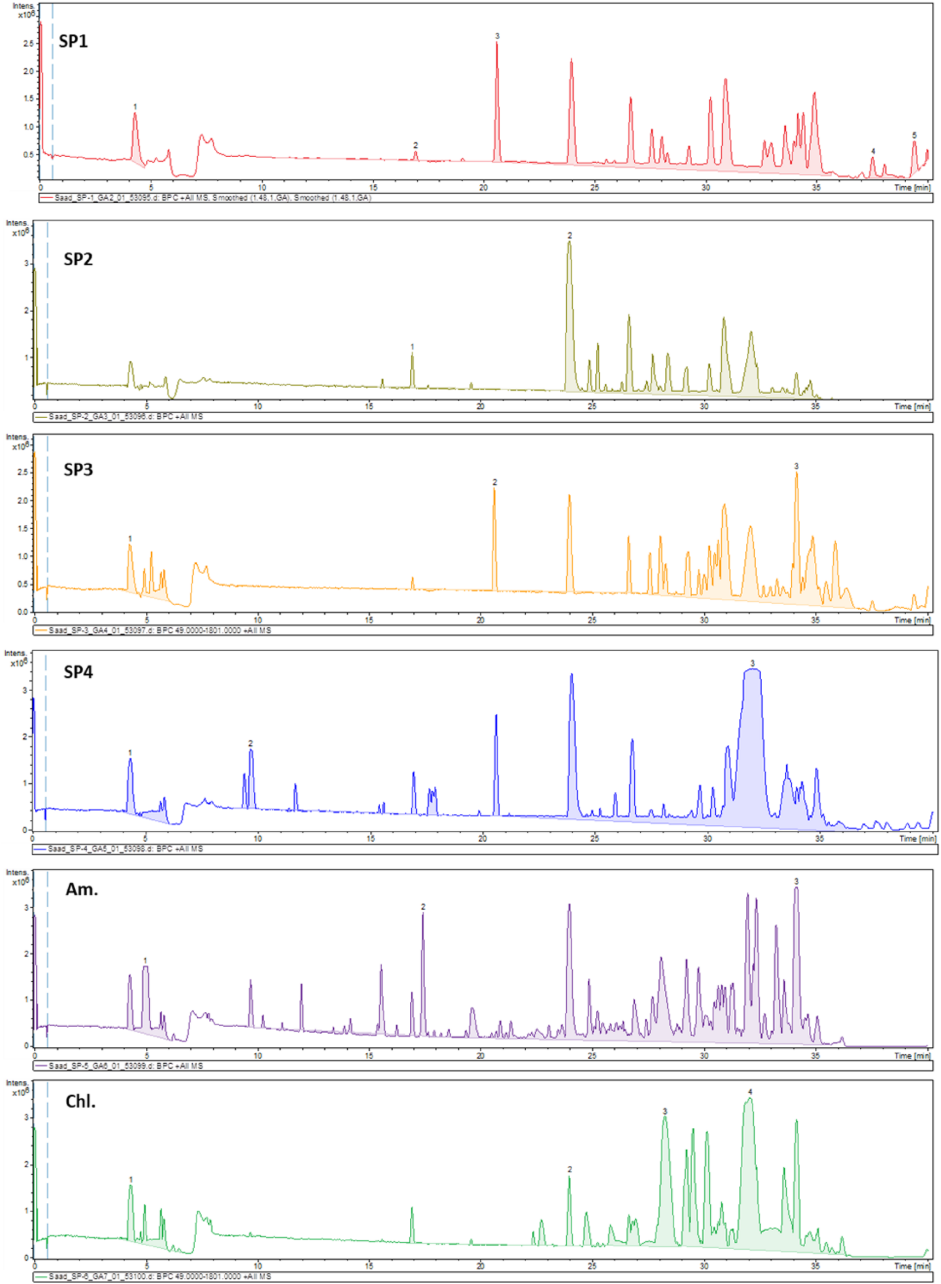
Base Peak Chromatogram (BPC) of the studied algal species in the positive ionization mode. Refer to Supplementary Table S3 for sample codes.

**Figure 3:**
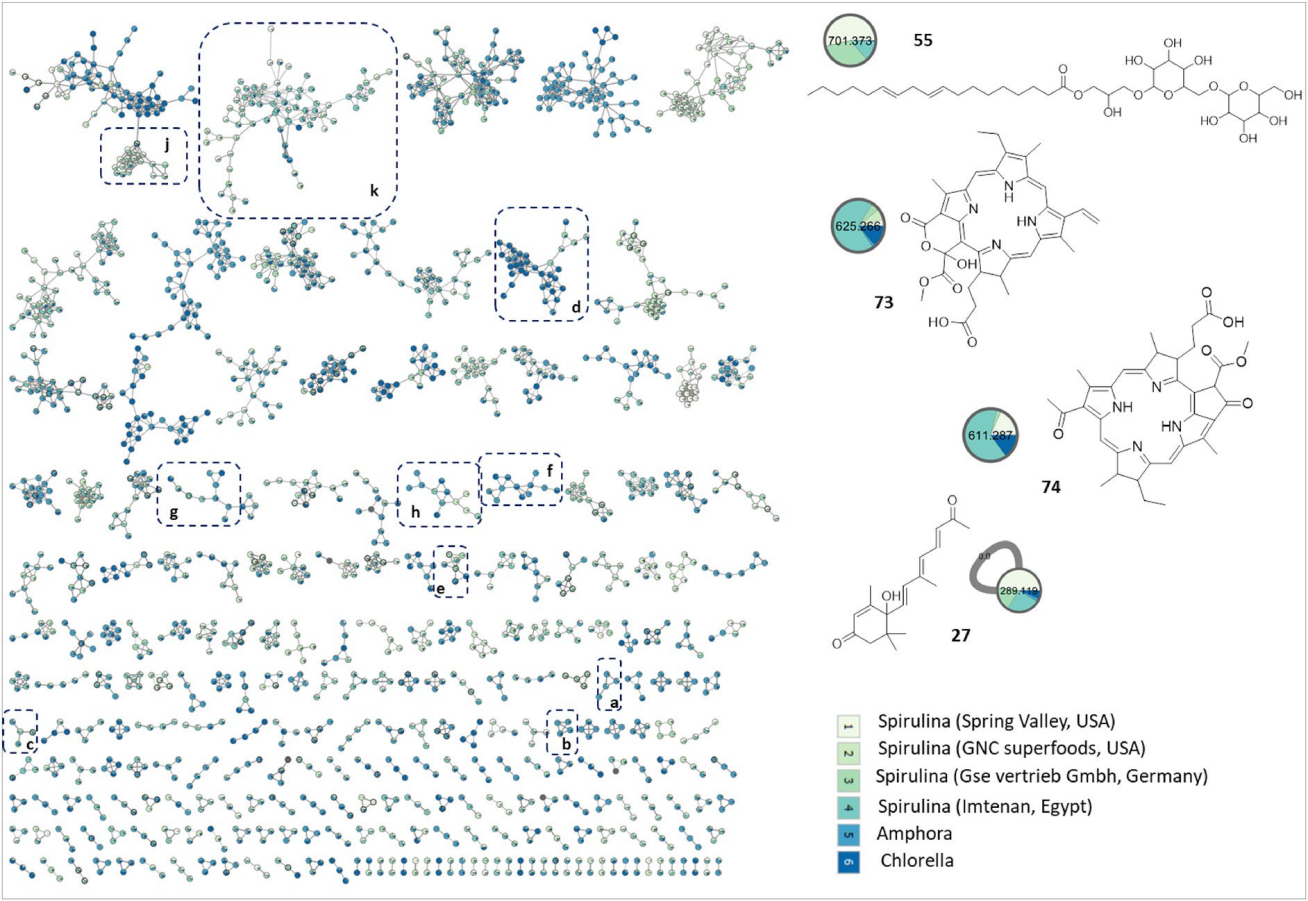
Feature-based Molecular Network (FBMN) of acquired UPLC-HRMS/MS data (positive ionization mode) of *Spirulina* samples *versus Amphora* and *Chlorella*. Clusters (**a**–**c**) fatty acids, (**d**) phospholipids, (**e**–**h**) nitrogenous lipids, (**j**) glycolipids, and (**k**) porphyrins.

UPLC-HRMS/MS in both ionization modes was employed for mapping of the selected algal species metabolome. The base peak chromatogram in both ionization modes showed a quite similar metabolome profile of the *Spirulina* samples, albeit being different from *Chlorella* and *Amphora* (Fig. 2, Supplementary Fig. 1). Subsequently, feature-based molecular network (FBMNs) was employed for a deeper comprehensive view of the chemical similarities and differences among the studied samples. The generated FBMNs offered a global overview of the existing chemical space and highlighted the similarities and differences among the selected algal species. The positive FBMN constituted 3798 nodes with 2046 connected features and the remaining as single nodes (Fig. 3). While the negative FBMN included 1411 features with 727 grouped nodes and the rest as singletons (Supplementary Fig. 2).

Annotation of the detected features relied on their elution order, chemical formulas, and their fragmentation pattern, synchronized with the FBMNs and the in silico fragmentation trees proposed by Sirius and searching literature^15,16^.

Visual exploration of chromatographic fingerprints among algal samples (i.e.* Spirulina*, *Amphora*, and *Chlorella*) delineated the abundance of fatty acids in *Amphora*, glycerolipids, glycolipids, and porphyrins in *Spirulina*, versus acyl amino acids enrichment in both *Amphora* and *Chlorella.* A total of 89 metabolites were annotated belonging to different classes, including fatty acids, phospholipids, glycolipids, glycerolipids, and porphyrins (chlorophyll pigments) (Supplementary Table S1).

#### Fatty acids

Microalgae are recognized as chief producers of a wide array of fatty acid derivatives such as hydroxyl fatty acids, oxylipins, alkenones, and polyols of potential nutraceutical and biotechnological applications^17^. For instance, unsaturated fatty acids (i.e., ω-3 fatty acids) represent an indispensable ingredient of healthy human diets, in addition to their potential to reduce the risks of atherosclerosis, cancer, and inflammation^17^. Recently, microalgae have attracted attention as an alternative and sustainable source of such nutritionally valuable molecules^18^.

Metabolome mapping of the selected algal specimens identified *Amphora* sp. as the most abundant in various types of fatty acids derivatives (i.e., hydroxylated fatty acids, keto fatty acids, and polyunsaturated fatty acids) as delineated from both FBMNs, positive FBMN (Fig. 2, clusters a–c), and negative (Supplemenatry Fig. 2, clusters a-g).

Annotated fatty acids included hydroxylated fatty acids (**1**–**7**, **12**, **18**, **19**, **22**), keto fatty acids (**8**, **10**, **17**, **20**, **21**), and unsaturated fatty acids (**13**, **15**, **16**) (Supplementary Table S1). Oxygenated fatty acids (i.e., hydroxylated and keto derivatives) showed the successive loss of H_2_O molecules [M-H-18]^−^, followed by consecutive losses of 14 Da indicative of the cleavage of the C–C bonds (Supplementary Figs. S3, S4). Currently, the widely consumed microalgae species include *Spirulina* (*Arthrospira* spp.) and *Chlorella* spp., and our profiling suggests that *Amphora* spp. presents a potential source of essential lipids, which may have a wide range of applications ranging from biofuels to nutraceuticals.

#### Phospholipids

Comparable to the abundant fatty acids in *Amphora*, phospholipids were present in the studied species, albeit with higher abundance in *Chlorella*. Two classes of phospholipids were detected in the studied algal samples exemplified by phosphocholines detected in the positive ionization mode (Fig. 3, cluster d), and phosphatidyl glycerols in the negative mode (Supplementary Fig. 2, cluster h) and highlighting the advantages for MS detection in different ion modes. Phosphocholines are characterized by the presence of fragment ions at *m/z* 184 and 104 corresponding to the phosphocholine and choline moieties^19^ (Supplementary Fig. S5). Detected phosphocholines included dioctanoyl phosphocholine (**28**), which grouped with other related unidentified analogs that have yet to be identified using other spectroscopic techniques post isolation.

Glycerophosphate lipids were distinguished by the loss of the two acyl moieties represented by several isomers of *O*-linoleoyl-*O*-stearoyl-sn-glycero-phosphate (**33**, **34**, and **35**) with an insignificant pattern of distribution among all algal samples (Supplementary Fig. S6).

#### Nitrogenous lipids

Several nitrogenous compounds were detected in positive ion mode, mainly sphingosines and acyl fatty acids (Fig. 3, clusters e–h) detected in both *Amphora* and *Chlorella* samples. The detected sphingosines produced a predominant fragment ion of [M + H-H2O]^+^ corresponding to the loss of a water unit, followed by the loss of another water moiety and formaldehyde, which results in the formation of highly abundant fragment [M + H-H_2_O-CH_2_O]^+^. Annotated sphingosines included aminohexadecanediol (**36**), aminotetradecanol (**37**), aminooctadecenediol (**38**), aminoheptadecanediol (**39**), aminoicosanetetrol (**40**), *N*-(dihydroxypropyl)octadecanamide (**43**), C20 sphingosine (**44**), and icosasphinganine (**45**) (Supplementary Fig. S7).

In addition to sphingosines, acylated amino acids were detected exemplified by *N-*palmitoyl-L-arginine (**42**) (Supplementary Fig. S8), detected in *Spirulina*, versus palmitoylputrescine (**47**), *N*-icosanoyl-glycine (**48**) in *Chlorella*, and octadecanoyl-*L*-carnitine (**49**) in both *Amphora* and *Chlorella*. Fatty acyl amides were also detected as linoleic acid amide (**41**) in *Chlorella* (Supplementary Fig. S9).

#### Phytoprostanes (Eicosanoids)

Phytoprostanes are produced through the autoxidation of α-linolenic acid under the influence of both biotic and abiotic factors. They are widely detected in terrestrial plants and scarcely reported in macroalgae^20^. Two phytoprostanes were detected in positive FBMN as self-looped nodes and were tentatively assigned as phytoprostane A1 (**51**), and deoxyphytoprostane (**52**), which have yet to be confirmed using other tools to be conclusive.

#### Glyco/ and glycerolipids

The positive FBMN unveiled the occurrence of glycolipids mainly as digalactosylacyl glycerols (DGG) and monogalactosyl acylglycerols (MGG) (Fig. 3, cluster j). As previously reported, glycolipids were detected chiefly as [M + Na]^+^ ions, which upon fragmentation, shows the loss of a hexosyl (− 162 Da), two hexosyls (-324 Da), and the loss of the fatty acyl chains as acid derivatives (–RCOOH)^21^.

Several DGG features were detected chiefly in *Spirulina* and were annotated as hydroxy-[(oxooctadecatrienyl)oxypropyl *O*-dihexoside (**54**), *O*- octadecaenoic-*O*-di-*O-*hexosyl-glycerol (**55**), and isomers of hexadecanoic acid-* O*-di-*O-*hexosyl-glycerol (**56** & **57**) (Supplementary Fig. S10). While MGG analogues were *O*-linolenoyl-*O*- hexosyl-glycerol (**58**), *O*-hexadecenoyl-hexosyl-glycerol (**59**), *O*-hexadecanoyl-hexosylglycerol (**57**), *O*-palmitoyl-*O*- hexosylglycerol (**62**) (Supplementary Fig. S11), and one diacyl MGG annotated as *O*-linoleoyl-*O*-palmitoyl-*O-* hexosyllglycerol (**63**).

Similar to the abundant glycolipids, several monoacylglycerols were also detected in *Spirulina* and *Chlorella*, mainly as singletons. Annotated features included monolinolenoyl glycerol (**64**), glyceryl palmitoleate (**65**), palmitoyl glycerol (**66**) (Supplementary Fig. S12), and isomers of linoleoyl glycerol (**67** and **68**).

#### Sulpholipids

Contrariwise to glycolipids abundance in positive FBMN, sulpholipids (i.e. sulphoquinovosyl monoacyl glycerols) were predominated in the negative FBMN (Supplemenatry Fig. 2, cluster J), specifically occurring in *Spirulina* samples. Sulphoquinovosyl monoacylglycerols are acylglycerols esterified with various fatty acids and a sulphoguinivose moiety (i.e. sulphonated hexose), which are better observed in the negative ionization mode owing to their strongly acidic sulphate group^22^.

Their MS^2^ spectra showed characteristic fragment ion at *m/z* 225 corresponding to [C_6_H_9_O_7_S]^-^ and the neutral loss of the fatty acyl side chain. Annotated sulphoquinovosyl monoacylglycerols included isomers of *O*-palmitoyl-*O-(*sulfo-galactosyl) glycerol (**86** and **87**) (Supplementary Fig. S13. Sulphoquinovosyl monoacylglycerols were previously detected in *Spirulina*^23^ and were isolated from the brown alga, *Ishige sinicola*, and exhibited algicidal activities^24^

#### Porphyrins

Porphyrins are chlorophyll pigments with a highly conjugated system showing a characteristic UV spectrum with λmax values at 450 and 680 nm^25^, (Supplementary Figs. S14, S15), and are known to occur in green algae^26^. Their identification is mainly aided by their distinctive UV spectrum, as they often fail to yield MS/MS fragments under the range of collision energy frequently set for small molecules^16^.

The positive FBMN revealed the abundance of porphyrins among examined *Spirulina* samples versus trace levels in *Chlorella* and *Amphora* (Fig. 3, cluster l), and suggestive that *Spirulina* presents a potential source of porphyrins. The MS^2^ spectra of porphyrins showed fragments resulting from the consecutive loss of H_2_O molecules, followed by the loss of CO and CH_2_ based on their substitutional pattern as previously reported^27,28^, (Supplementary Figs. S14, S15).

The annotated porphyrins included bacteriopheophorbide a (**74**), mesoporphyrin IX (**77**), harderoporphyrin (**78**), methyl 8-ethyl-12-methylbacteriopheophorbide c (**81**), tetramethyl-3-(methoxycarbonyl)-5-(1,2-dioxo-2-methoxyethyl)-13-vinyl-18-ethyl-7,8-dihydro-21H,23H-porphyrin-7-propionic acid (**82**), hydroxy pheophorbide a (**83**), and pheophorbide a (**84**). It is worth mentioning that along the known porphyrins, several unknown analogs were observed within the same cluster (Fig. 3, cluster l).

### Primary metabolites profiling of *Spirulina* samples versus *Amphora* and *Chlorella* via GC–MS post-silylation

For an overview of the constitutive primary low molecular weight metabolites of the examined microalgal samples, GC–MS post-silylation was employed to better assess its nutritive value and to complement UPLC-MS results. A total of 56 metabolites (Supplementary Table S2) were identified, including alcohols, amino acids, fatty acids/ esters, glycosides, hydrocarbons, inorganic compounds, nitrogenous compounds, organic acids, sugars, sugar alcohols, and terpene alcohols, as detailed in the next subsections.

#### Fatty acids/esters

GC–MS analysis of the examined algal samples delineated the abundance of fatty acids/ esters, including saturated and unsaturated fatty acids (i.e. ω-3, ω-6, and ω-9), in algal species (14- 38%), thus representing a potential food supplement. Among the annotated fatty acids/ esters, palmitic acid (**17**) was the predominant form in *Spirulina* and *Amphora versus* glyceryl monostearate (**25**) in *Chlorella*. Other saturated fatty acids/ esters included stearic acid (**21**), arachidic acid (**22**), 2-palmitoyl glycerol (**23**), and 1-monopalmitin (**24**). With regards to unsaturated fatty acids, linoleic acid (**18**) was the most abundant in all samples, followed by linolenic (**18**) and oleic acid (**19).** Linoleic acid is evidenced to exert beneficial actions on blood lipids and lower serum cholesterol and blood pressure. Further, The nutritional value of linoleic acid is metabolized metabolism at tissue levels to produce the hormone-like prostaglandins^29^.

#### Glycosides

In contrast to the abundant fatty acids in all samples, glycerol glycosides were the second dominating metabolite class in *Spirulina* (24–40%) samples occurring as glycerol glycosides (**26**, **27** &** 28**), and in accordance with UPLC-HRMS/MS findings (Fig. 3, Supplementary Fig. 2).

#### Sugars

Following the abundant fatty acids and their acyl derivatives, *Spirulina* samples showed much higher sugar levels (9–27%) compared with *Amphora* (2%) and *Chlorella* (14%). The predominant sugars in *Spirulina* were 3-mannobiose (**51**), followed by melibiose (**54**) and sucrose (**53**). For *Amphora*, sucrose (**53**) and melibiose (**54**) were the most abundant forms *vs.* sucrose (**53**) and glucose (**50**) in *Chlorella*. Lastly, the sugar alcohol; myo-inositol was detected at trace levels in all samples and suggestive that disaccharides account more for examined microalgal sugar profile and likely taste.

Mannobiose is well reported to act as an immunostimulatory molecule in murine dendritic via stimulating cytokine production in RAW264.7 macrophages^30^. Whereas, melibiose exerts immunostimulatory and anti-allergic actions and enhances minerals absorption, promoting the growth of beneficial gut microbiota, especially *Bifidobacterium* and *Lactobacillus* strains^31^. The effecot fo algal products as prebiotics should be encouraged based on these detailed chemical analyses.

#### Amino acids/ nitrogenous compounds

In contrast to the abundance of sugars in *Spirulina* samples, *Amphora* and *Chlorella* showed higher levels of free amino acids (28% and 17%, respectively). *Chlorella* and *Amphora* were specifically enriched with essential amino acids such as valine (**5**), leucine (**6**), isoleucine (**7**), and allo-isoleucine (**8**). These essential amino acids add to the high nutritional value of microalgae and promote their use as supplements or nutraceuticals^32^. While non-essential amino acids included alanine (**4**), serine (**9**), asparatic acid (**10**), L-5-oxoproline (**11**), and glutamic acid (**12**).

Besides being enriched with amino acids, other nitrogenous compounds abundantly observed in *Amphora* and *Chlorella* (i.e., at 14 & 12%, correspondingly) including niacin (**33**) as the most prominent one. Other nitrogenous compounds included uracil (**35**), adenine (**36**), and adenosine (**37**).

#### Organic acids & alcohols

Organic acids showed comparable levels (7–14%) among examined microalgae, with caproic acid (**40**) as the major form (≈ 5–8%) in all the studied samples. Other organic acids included 3-hydroxybutyric acid (**44**), succinic acid (**38**), lactic acid (**39**), glycolic acid (**40**), and pyruvic acid (**41**). These organic acids are important intermediates of the tricarboxylic acid (TCA) cycle contributing to energy production and to the biosynthesis of more valuable metabolites such as lipids, proteins, and pigments^33^.

*Amphora* and *Chlorella* samples showed higher alcohol levels (6% and 4%) compared with *Spirulina* samples (0.6–1.5%), exemplified by 2,3 butandiol (**1**) and glycerol (**2** and **3**).

#### Miscellaneous

Other metabolites included 1,2,4-benzenetriol (**47**) as the only detected phenolic specially enriched in *Spirulina* samples (0.7–2.8%). While phytol (**56**); a terpene alcohol, was detected in all samples. Aliphatic hydrocarbon was represented by 2,6,10-trimethyl- pentadecane (**29**), and phosphoric acid (**30** and **31**) as the only inorganic acid detected.

### Multivariate unsupervised HCA and PCA analysis of GC–MS dataset

Multivariate analysis was further conducted via unsupervised pattern recognition methods i.e., principal component analysis (PCA), hierarchical cluster analysis (HCA) using GC–MS data matrix to establish a metabolite-based clustering of the three algal species and assess their metabolome similarity and/or heterogeneity^34^.

**Figure 4:**
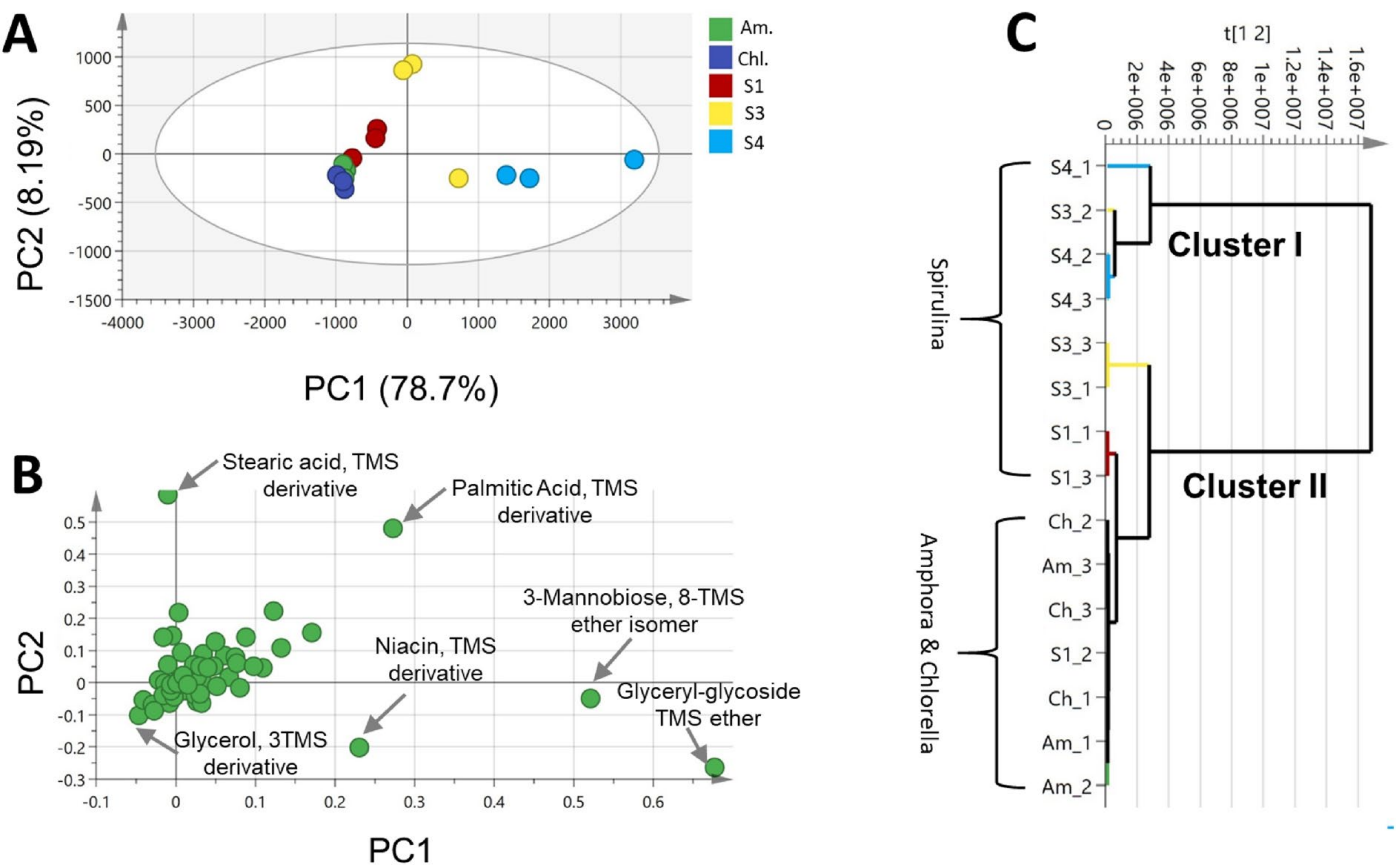
Principal component analysis (PCA) and hierarchical clustering analysis (HCA) of GC–MS dataset of *Spirulina* (S1, S3 & S4), *Amphora* (Am.), *Chlorella* (Chl.). (**A**) Score plot of PC1 vs. PC2 scores. (**B**) Loading plot for PC1 & PC2 contributing metabolites and their assignments. The metabolome clusters are located at the distinct positions in two-dimensional space described by two vectors of principal component 1 (PC1) and principal component 2 (PC2) = 78.7% and PC2 = 8.2%. (C) HCA plot. Refer to Supplementary Table S3 for sample codes.

PCA modeling of GC–MS dataset of algal samples (S1, S3, S4, Am., Chl.) was performed with the two principal components (PCs), explaining 87.7% and 8.2% of the total variance, respectively. PCA score plot (Fig. 4A) showed grouping of one sample of S3 along with S4 samples to the right-hand side of the plot; however, a separate group of *Spirulina* and *Amphora* and *Chlorella* (S1, Am. and chl.) samples was located towards the left side of the plot. Loading plot (Fig. 4B) explained such clustering owing to the abundance of both *Spirulina* samples (S3 & S4) in palmitic acid, mannobiose, glyceryl-glycoside, and niacin, versus glycerol enrichment in *Spirulina* sample (S1), *Amphora* (Am.), and *Chlorella* (chl.).

Hierarchical cluster analysis (HCA) further provided sample grouping in the form of a dendrogram encompassing two main clusters (I & II); cluster (I) contained majorly *Spirulina* samples (S1, S3 and S4), and cluster (II) included *Amphora* and *Chlorella* samples along with one *Spirulina* sample (S1) (Fig. 4C). Accordingly, *Spirulina* (S1) sample was omitted from subsequent modeling.

### Supervised OPLS-DA analysis of GC–MS dataset

**Figure 5:**
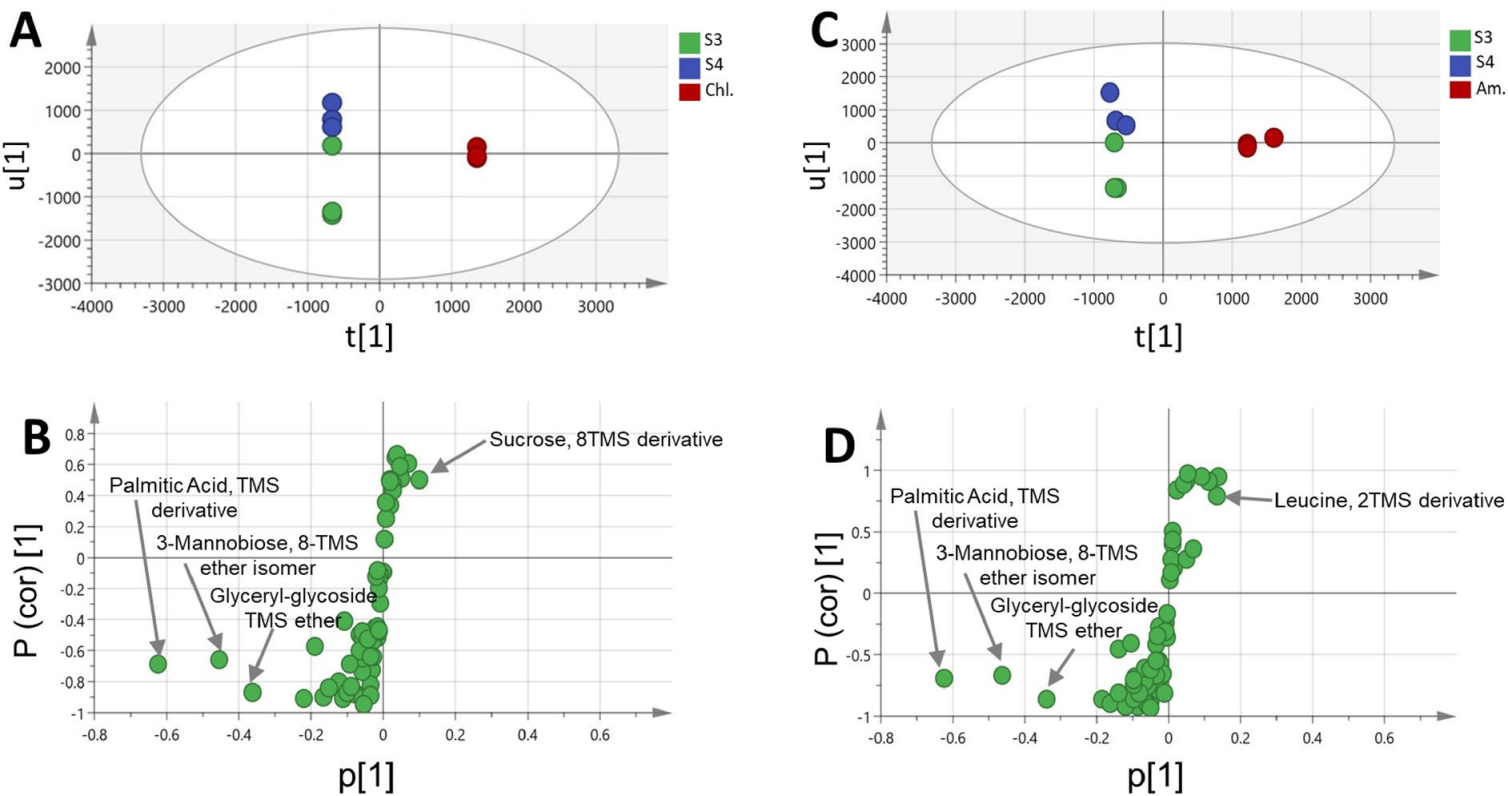
OPLS score plot derived from GC–MS dataset modelling of *Spirulina* (S3 and S4) samples against *Chlorella* (Chl.) and *Amphora* (Am.) samples each at a time (**A**,**C**). Their corresponding S-plots (**B**,**D**) show the covariance p^1^ against the correlation p(cor)^1^ of the variables of the discriminating component of the OPLS-DA model. Cut-off values of P < 0.05 were used,selected variables are highlighted in the S-plot with identifications are discussed in text. Refer to Supplementary Table S3 for sample codes.

In order to achieve a clear separation among samples and aid in identifying potential compositional markers, orthogonal projection to latent structures-discriminant analysis (OPLS-DA) of the GC–MS dataset was further attempted. When *Spirulina*, *Amphora* and *Chlorella* samples were modeled against one another using OPLS-DA, no separation between the samples was achieved in the derived score plot (Supplementary Fig. S16A). Hence, another OPLS-DA model was employed by modeling GC metabolite profiles of *Spirulina* samples (S3 and S4) against *Chlorella* (Chl.), and *Amphora* (Am.) samples each at a time (Fig. 5A–D). A better separation was obtained in both models, which explained 96 and 98% of the total variance (R2 = 0.99 and 0.98) with the prediction goodness parameter Q2 = 0.87 and 0.85, respectively (Supplementary Figs. S17B, S18B). Other validation parameters were calculated for both models including permutation tests that showed negative Q2 intercept value and CV-ANOVA with p value below 0.05 (Supplementary Fig. S17 and S18C,E, respectively) typical for valid models. The model classifcation ability was tested by ROC curve (Supplementary Figs. S17, S18D, respectively), which showed area under the ROC curve (AUC) of 1.0 indicating an efective classifcation model. The S-plot (Fig. 5B,D) showed that palmitic acid, mannobiose, and glyceryl-glycoside were more abundant in *Spirulina* and in agreement with PCA results. In contrast, *Chlorella* was more enriched in sucrose, whereas, *Amphora* was more rich in leucine.

### UV/VIS fingerprinting assisted by multivariate analysis

As revealed from the UPLC-HRMS/MS analysis, metabolites with active UV chromophores were detected (i.e. porphyrins and, to less extent, carotenoids) in the studied microalgal species. Accordingly, UV–Vis spectrophotometric analysis was adopted to offer a rapid and affordable analysis when compared to other hyphenated techniques (i.e. UPLC-HRMS/MS and GC–MS), to examine its classification potential of the examined microalgal samples. Representative UV–Vis spectra of *Spirulina*, *Amphora,* and *Chlorella* samples are shown in Supplementary Fig. S19. The spectra displayed UV maxima at wavelengths of 210 nm and 400–410 nm due to the absorbance of carotenoids, in addition to UV maxima at wavelengths of 650–670 nm corresponding to the absorbance of porphyrins^35^.

Carotenoids act as provitamin A precursors being converted inside the human body into vitamin A. Zeaxanthin, lutein, and β-carotene are the carotenoids previously identified in *Spirulina*. Lutein and zeaxanthin exhibit a protective action against visual disturbances and cognitive diseases. Chlorophyll is another chief green pigment present in *Spirulina* that has been characterized for its antimutagen, chemoprotective, antioxidant, anti-inflammatory, and antimicrobial properties^35^.

In our study, *Spirulina* was found to be more enriched in chlorophyll pigments (i.e., porphyrin) which are evident from the higher absorbance at wavelengths of 400–430 nm than that of *Chlorella* and *Amphora* samples. *Spirulina* species were formerly reported to be distinguished from the *Chlorella* by their higher content of carotenoids and chlorophyll pigments^35^.

### Multivariate data analyses of *Spirulina*, *Chlorella*, and *Amphora* via UV/VIS spectral fingerprints

**Figure 6:**
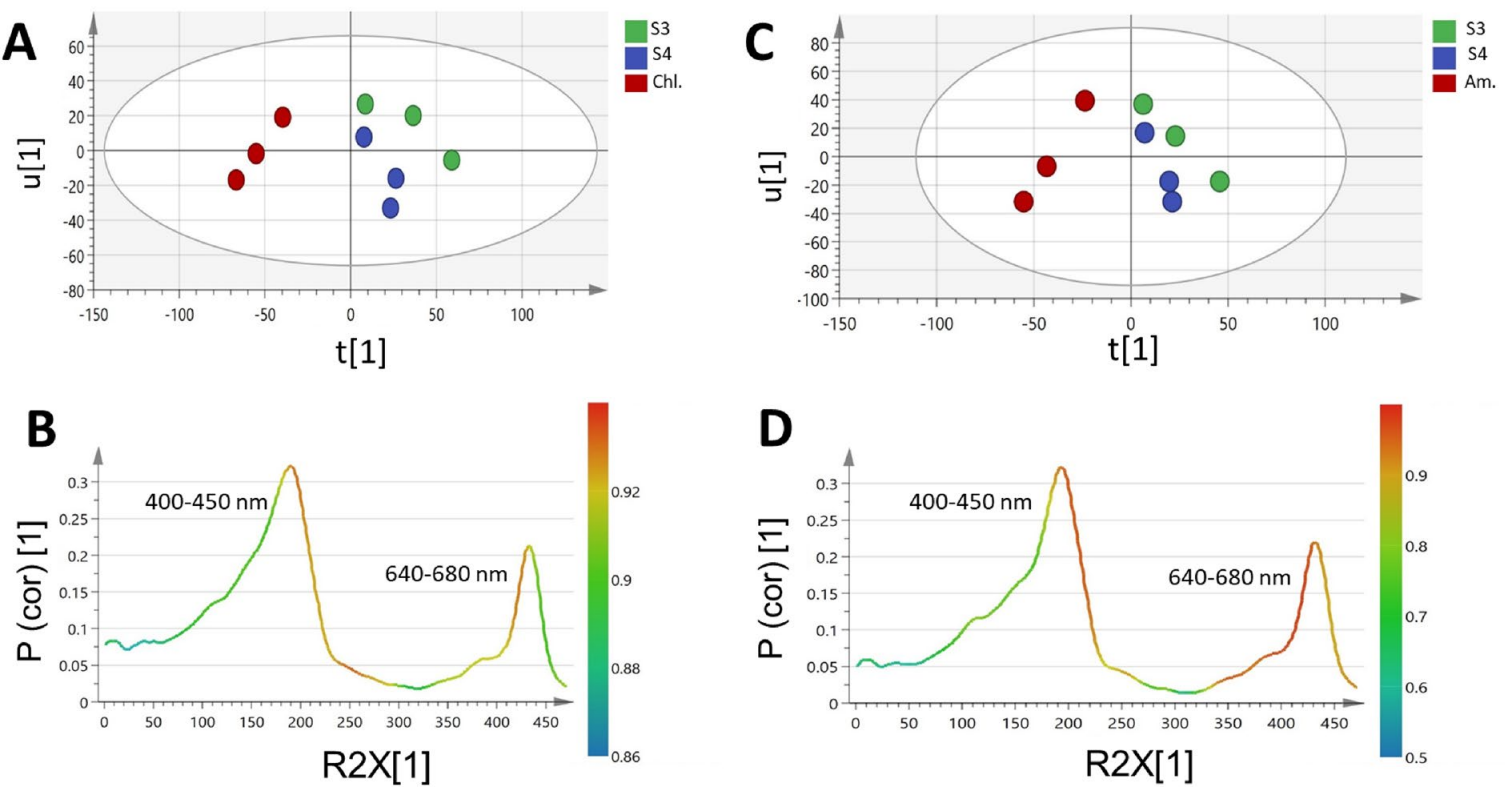
OPLS score plot derived from UV–Vis dataset modelling of *Spirulina* (S3 & S4) samples against *Chlorella* (Chl.) and *Amphora* (Am.) samples each at a time (**A**,**C**). Their corresponding S-line plots (**B**,**D**) show the covariance p^1^ against the correlation p(cor)^1^ of the variables of the discriminating component of the OPLS-DA model. Cut-off values of P < 0.05 were used,selected variables are highlighted in the S-plot and discussed in text. Refer to Supplementary Table S3 for sample codes.

For the sake of selecting the most discriminative wavelengths in the separation among algal species under study, PCA analysis of their UV/VIS fingerprints was carried out (Supplementary Fig. S18). A total variance of 99.7% was explained by the first two components, though the model did not show a clear segregation between the three species as depicted in the PCA score plot (Supplementary Fig. S20A). Accordingly, supervised OPLS-DA was further employed to model UV/VIS fingerprints of *Spirulina* samples against *Chlorella* and A*mphora* samples, each at a time to build two classification models for the discrimination between the three species (Fig. 6).

Both OPLS-DA models (Fig. 6) showed a good classification performance, as implied by R2 and Q2 (0.85 and 0.54) and (0.83 and 0.68), respectively. The models were validated using permutation test with 200 times and CV-ANOVA which demonstrated that the model is not over fitted (Supplementary Figs. S21, S22C,E, respectively), with negative Q2 intercept value and p value below 0.05. ROC curve obtained for both models had an AUC of 1, which is indicative of an efective classifcation model (Supplementary Fig. S21, S22D, respectively).

The derived score plot (Fig. 6A,C) showed a clear separation between *Spirulina* and the other microalgae species (i.e.,* Chlorella* and *Amphora*), respectively. The UV/VIS spectral regions of 400–430 nm (corresponding to carotenoids absorbance) and 650–680 nm (due to chlorophyll absorbance) were identified by the S-line plot of loadings (Fig. 6B,D) as the most discriminative wavelengths between the samples in both models.

## Discussion

Microalgae represent a potential source of nutraceuticals and important bioactive metabolites. In the current study, we aimed to map the metabolome of different commercial *Spirulina* samples in comparison to *Chlorella* and *Amphora*, via different metabolomics technologies (i.e., UPLC-HRMS/MS and GC–MS post-silylation). UPLC-HRMS/MS analysis allowed for exploring their large molecular weight metabolome, such as polar lipids, including phospholipids, nitrogenous lipids, glycerolipids, and glycolipids, in addition to free fatty acids, and pigments.

*Spirulina* samples were enriched in glycolipids (i.e. digalactosylacyl glycerols) as the predominant lipid class, with the occurrence of few sulphoquinovosyl monoacylglycerols, *versus* the abundance of phospholipids in *Chlorella*. Similarly, *Amphora* was proven as a rich source of nutritionally important fatty acids supporting its potential use as a biofuel and food supplement, agreeing with previous studies^12^. Glycerolipids are reported to exert anti-inflammatory properties^12^, sulpholipids to exhibit antiviral, anticancer, immunsuppressive and anti-inflammatory properties^23^, while phospholipids are recognized as an excellent source of polyunsaturated fatty acids targeting the brain thus improving memory in elderly and neurological patients^12^. Owing to these nutraceutical and pharmaceutically significant ingredients, there is a growing trend in the incorporation of microalgal biomass in novel cuisine^3^.

Similarly, the main pigments detected were porphyrins chiefly among the examined *Spirulina* samples and sporadically in *Chlorella* and *Amphora.* These findings contradict previous reports in which higher amounts of these photosynthetic pigments were observed in *Chlorella* in comparison to *Spirulina* supplements^36^, which may be attributed to the variation in the cultivation conditions and extraction protocols. These green-colored pigments are valued not only as food colorants but also as beneficial ingredients for their antimutagenic, antioxidant, chemopreventive, and antimicrobial activities^37^.

Comparably, GC–MS analysis was recruited for cataloging their low molecular weight primary metabolites and to highlight their nutritive value, and delineated fatty acid/ esters and glycosides as the most predominant and following UPLC-HRMS/MS results, though revealing other markers. For instance, GC–MS analysis delineated higher content of essential amino acids in *Chlorella* and *Amphora*, contributing to their high nutritional value and advocating their use as nutraceutical and nutritional supplements.

For the discrimination of the studied microalgal species, multivariate data analysis of the GC–MS metabolite profiles showed that palmitic acid, mannobiose, and glyceryl-glycoside were more abundant in *Spirulina* samples. In contrast, *Amphora* and *Chlorella* were more enriched in leucine and sucrose, respectively.

For their routine discrimination, UV/VIS spectral fingerprints were efficient in distinguishing between samples based on their pigment composition, serving as a rapid tool for routine quality control. UV absorbance of porphyrins and carotenoid pigments at wavelengths of 400–430 nm and 650–680 nm were revealed by modeling of the UV/Vis spectral fingerprinting as being the most differentiating wavelengths.

Conclusively, both UPLC-HRMS/MS and GC–MS analyses revealed the studied microalgal species as valuable sources of essential lipids. Yet considerable variations in metabolome were observed among the *Spirulina* samples, which could be attributed to differences in cultivation settings, which warrants the need to optimize the cultivation conditions to unrestrictedly promote their use as dietary supplements and to prevent the growth of undesired organisms. Additionally, more information is needed about the safety of microalgae as nutraceuticals for their possible contamination with cyanotoxins, heavy metals, and pesticides in the future. The enrichment of *Spirulina* in several metabolites of a potential prebiotic nature warrants its inclusion in nutraceuticals to serve for improving gut functions.

## Materials and methods

### Microalgae samples

The study included four *Spirulina platensis* samples (S1, S2, S3 and S4) procured from USA, Germany, and Egypt, in addition to two allied species, namely *Amphora *(Am.) and *Chlorella* (Chl.), which were kindly obtained from National Research Center, Dokki, Egypt. Sample codes are listed in Supplementary Table S3.

### Chemicals and materials

All chemicals and standards were purchased from Sigma-Aldrich (St. Louis, MO, USA). MilliQ water was supplied by a Millipore MR3 purifier system and was used for UPLC-HRMS/MS analysis.

### Extraction and sample preparation for UPLC-HRMS/MS

The powdered samples (5 g) were extracted by soaking in 100 ml of 100% methanol for 48 h. The extract was then filtered and evaporated under reduced pressure at 40 °C. The obtained extracts were further purified using Strata® Silica-Based Solid Phase Extraction (SPE) column (Phenomenex, CA, USA) with gradient 0∼100% methanol /water and 100% methanol as final elution to reduce the chlorophyll content. Collected fractions were then filtered, evaporated under reduced pressure, and finally lyophilized and kept frozen at − 20 °C.

### UPLC-HRMS/MS analysis

MaXis-4G instrument (Bruker Daltonics, Bremen, Germany) attached to an Ultimate 3000 HPLC (Thermo Fisher Scientific) was used for HR-MS analysis. The HPLC method was (0.1% FA in H_2_O as solvent A and MeOH as solvent B), an isocratic gradient of 10% B for 10 min, 10% to 100% B in 30 min, 100% B for an additional 15 min, using a flow rate of 0.3 ml/min; 5 µl injection volume and UV detector (UV/VIS) wavelength monitoring at 336, 280 and 238 nm. The separation was performed on a Nucleoshell 2.7 mm 150 × 2 mm column (Macherey–Nagel), and the range for MS acquisition was *m/z* 50–1800. A capillary voltage of 4500 V, nebulizer gas pressure (nitrogen) of 2 (1.6) bar, ion source temperature of 200 °C, a dry gas flow of 9 l/min source temperature, and spectral rates of 3 Hz for MS^1^ and 10 Hz for MS^2^ were used. For MS/MS fragmentation, the 10 most intense ions per MS^1^ were chosen for subsequent CID with stepped CID energy applied. The employed parameters for tandem MS were applied following^38^.

### Feature-based molecular networking and compounds annotation

Raw data inspection was performed using Compass Data Analysis 4.4 (Bruker Daltonics®). Metaboscape 3.0 (Bruker Daltonics®) was utilized for feature detection, grouping, and alignment, employing the T-ReX 3D (Time aligned Region Complete eXtraction) algorithm^39^.

Bucketing was performed with an intensity threshold of 10 × 10^4^ and 10 × 10^3^ for the positive and negative modes, respectively. The retention time range was from 0.5 to 40 min with a restricted mass range *m/z* from 190 to 1800. The produced MGF files and the feature quantification tables (CSV file) were used to construct two feature-based molecular networks (FBMNs) for both ionization modes, following the online workflow in the GNPS platform (http://gnps.ucsd.edu)^40^. The parameters applied for the construction of the FBMNs via the GNPS platform are detailed in Supplementary Table S4.

Cytoscape version 3.7.1.60 was used for the network visualization. Sirius + CSI: FingerID 5.6.3 was used for the manual putative structures identification^41^, assisted by the molecular formula prediction and candidate search with *m/z* tolerance set to 20 ppm connected to online Pubchem^42,43^.

### GC–MS analysis of silylated primary metabolites

The finely ground algal powders (100 mg) were extracted with 100% methanol (5 ml) with sonication with frequent vortex shaking. The obtained extracts (100 µl) were aliquoted in screw cap vials and evaporated under a stream of nitrogen gas until complete dryness. Derivatization and GC–MS analysis were performed following a previously described protocol^44^.

Metabolite identification was based on their Kovat retention indices (KI) relative to C6-C20 n-alkane series and spectral matching with the NIST and WILEY libraries. Peaks deconvolution was performed through AMDIS software (http://www.amdis.net) before the mass spectral matching, and MS-Dial was used for the extraction of the peaks abundance data following^45^.

### UV/VIS fingerprinting

For UV fingerprinting, 200 µl of each extract in MeOH were pipetted into microplate wells (n = 4) of the 96-well quartz cell of the Gen 5 UV/Vis microplate reader (BioTek Instruments, Inc., Winooski, VT, USA). The absorption spectra were recorded in the range of 200–800 nm following the exact procedure described in^14^.

### Multivariate data analysis (MVA) of GC–MS and UV spectral datasets

MVA was employed to highlight differences and similarities of different commercial *Spirulina* samples together with *Amphora* and *Chlorella* samples in an untargeted manner and to aid in identifying markers for each. The normalized GC–MS data matrix to spiked standard internal xylitol was modeled using unsupervised pattern recognition methods i.e. principal component analysis (PCA) and hierarchical cluster analysis (HCA), as well as supervised method i.e. orthogonal projection to latent structures-discriminant analysis (OPLS-DA). PCA was implemented to visualize spacing between the samples as an unsupervised model, while OPLS-DA served as a discriminatory tool to identify how *Spirulina* samples are discriminated from each other and *Amphora *and *Chlorella*. Variations between sample sets were demonstrated using a score plot either in PCA or OPLS-DA model using SIMCA software (version 14.1) as in^14^. In addition, permutation tests and CV-ANOVA and ROC curve were used to validate the results of the developed OPLS-DA models.

Likewise, UV/Vis spectral data matrix exported using Excel (Excel 2016, Microsoft®, Redmond, WA, USA) for all samples, including their replicates, were mean-centered and Pareto scaled for variables representing absorbance readings between 200 and 650 nm. The data set was then modeled, similar to GC–MS, using PCA and OPLS-DA models. The OPLS-DA models were calculated using the default seven-fold cross-validation method yielding acceptable R2X, R2Y, and Q2 with no negative values and values for both above 0.5. The same validation procedure was performed as in the GC–MS dataset to ensure no model overfitting.

### Supplementary Information

Supplementary Information.

## Data Availability

All data generated or analysed during this study are included in this published article (and its Supplementary Information files).
