# Supplementary material for: A multiplex metabolomic approach for quality control of Spirulina supplement and its allied microalgae (Amphora & Chlorella) assisted by chemometrics and molecular networking
Source: Sci Rep. 2024 Feb 2;14:2809. doi: 10.1038/s41598-024-53219-5 (PMC10837195; doi:10.1038/s41598-024-53219-5)
Supplement: Supplementary file 1 — Supplementary Information. [file 41598_2024_53219_MOESM1_ESM.pdf]

iA Multiplex Metabolomic Approach for Quality Control of Spirulina Supplement and its Allied Microalgae (Amphora & Chlorella) Assisted by Chemometrics and Molecular Networking

Nesrine Hegazi[a], Amira R. Khattab[b], Hamada H. Saad[a], Bishoy Abib[d], Mohamed A. Farag [e*]

[a]Department of Phytochemistry and Plant Systematics, National Research Centre, Dokki, Cairo 12622, Egypt
[b]Pharmacognosy Department, College of Pharmacy, Arab Academy for Science, Technology and Maritime Transport, Alexandria 1029, Egypt
[c]Chemistry department, American University in Cairo, Cairo, Egypt
[d]Pharmacognosy Department, College of Pharmacy, Cairo University, Kasr El Aini St., P.B. 11562, Cairo, Egypt

***Corresponding author: Nm.hegazi@nrc.sci.eg (N. Hegazi), Mohamed.farag@pharma.cu.edu (M.A. Farag).**

Supplementary Data

Supplementary Table S1: Metabolites identified in micro-algal samples (*Spirulina* (S1-S4), *Amphora* (AM.), and *Chlorella* (Chl.)) *via* UPLC-HRMS/MS analysis. -:absent, tr: traces, +: present, ++: abundant, +++: major.

	Compound name	RT	M-H (error in ppm)	MS^2	M+H (error in ppm)	MS^2	Formula	S1	S2	S3	S4	Am.	Chl.	Reference
	**Fatty acids**													
1.	Hydroxyoctanedioic acid	11.04	189.0770 (-0.7)	143, 127			$C_8H_{14}O_5$	-	-	tr	tr	++	tr	
2.	Hydroxydecanedioic acid isomer I	13.55	217.1082 (-0.3)	157			$C_{10}H_{18}O_5$	tr	tr	tr	tr	+++	tr	
3.	Hydroxydecanedioic acid isomer II	14.53	217.1084 (-1.3)	199, 171, 155			$C_{10}H_{18}O_5$	tr	tr	tr	tr	+++	tr	
4.	Tetrahydroxy-octadecadienoic acid	15.29	343.2127 (-0.4)	325, 307, 299, 289, 271, 229, 211, 183, 171			$C_{18}H_{32}O_6$	-	-	-	tr	+++	+	
5.	Trihydroxyhexadecenoi c acid	17.32	301.2018 (0.8)	283, 265., 201, 183, 143			$C_{16}H_{30}O_5$	-	-	-	tr	+++	tr	
6.	Hydroxydodecandioic acid	17.62	245.1392 (1.0)	217, 199, 171, 155, 125			$C_{12}H_{22}O_5$	-	-	-	-	++	-	
7.	Trihydroxyoctadecenoic acid	19.54	329.2336 (-0.7)	229, 211, 171			$C_{18}H_{34}O_5$	tr	tr	-	tr	+++	tr	
8.	Oxo-octadecadienoic Acid isomer I	19.63			295.2264 (1.2)	277, 259, 249, 241, 179,165,	$C_{18}H_{30}O_3$	tr	+	-	+	+++	+	GNPS

	Compound name	RT	M-H (error in ppm)	MS²	M+H (error in ppm)	MS²	Formula	S1	S2	S3	S4	Am.	Chl.	Reference
						151, 133, 125, 107, 95								
9.	Dodecandioic acid	21.37	229.1444 (0.4)	211, 167, 130			$C_{12}H_{22}O_4$	tr	tr	tr	tr	+++	+	
10.	Oxo-octadecatrienoic acid	21.53			293.2109 (0.9)	275, 245, 163, 151, 133, 119, 105, 95, 81, 67	$C_{18}H_{28}O_3$	+	+	+	++	+++	+	GNPS
11.	Hydroxy-oxo-octadecadienoic acid	21.54			311.2213 (1.3)	293, 275, 245, 223, 198, 181, 163, 151, 133, 119, 107, 93, 81, 67	$C_{18}H_{30}O_4$	tr	+	tr	+	+++	+	
12.	Trihydroxyoctadecadienoic acid	21.55	327.2173 (1.1)	309, 291, 209, 197, 185, 171, 155			$C_{18}H_{32}O_5$	tr	tr	tr	tr	+++	tr	
13.	Hexadecatetraenoic acid	21.79			249.1849 (0.2)	232, 214, 189, 161, 147, 133, 119, 105, 93, 79, 67	$C_{16}H_{24}O_2$	-	-	tr	tr	+++	-	
14.	Undecanedioic acid	19.72	215.1291 (-0.8)	197, 153			$C_{11}H_{20}O_4$	tr	tr	tr	tr	+++	+	GNPS
15.	Octadecatrienoic acid,	23.46			279.2317 (0.6)	263, 235, 189, 165, 149, 135, 121, 107, 95, 81, 67	$C_{18}H_{30}O_2$	-	tr	-	++	+++	-	
16.	Octadecatetraenoic acid	24.00			277.2161 (0.4)	260, 243, 171, 163, 147, 135,	$C_{18}H_{28}O_2$	tr	-	-	-	+++	-	[1]

	Compound name	RT	M-H (error in ppm)	MS²	M+H (error in ppm)	MS²	Formula	S1	S2	S3	S4	Am.	Chl.	Reference
						121, 107, 93, 81, 67								
17.	Oxo-octadecadienoic Acid isomer II	24.06			295.2266 (0.6)	277, 259, 249, 241, 179, 165, 151, 133, 125, 107, 95	$C_{18}H_{30}O_3$	tr	+	+	+	+++	-	GNPS
18.	Hydroxy-(hydroxymethyl) -trimethyl-tetradecadiene dioic acid	24.10	341.1971 (-0.5)	309, 291, 269, 195, 177, 165, 151, 135, 125, 109, 93, 83, 69			$C_{18}H_{30}O_6$	-	tr	-	-	-	-	
19.	Trihydroxyoctadecatrienoic acid	24.27	327.2178 (-0.4)	291¸ 273, 247, 221, 207, 197, 181, 163, 153, 137, 125			$C_{18}H_{32}O_5$	-	tr	-	tr	-	tr	[2]
20.	Oxohexadecenoic acid	24.40			291.1951 (1.1)	275, 257, 245, 229, 205, 191, 179, 161, 151, 133, 119, 105, 93, 81, 71	$C_{18}H_{26}O_3$	-	+	+	tr	+	tr	
21.	Oxo-octadecatrienoic acid	24.88			293.2109 (0.7)	275, 247, 223, 205, 177, 163, 151, 137, 119, 107, 95, 81, 67	$C_{18}H_{28}O_3$	+	+	+	+	++	+	
22.	Tetrahydroxy-octadecadienoic acid	25.02	343.2129 (-0.7)	309, 291, 273, 247, 209, 197, 183, 151, 137, 125, 109, 95, 83, 69			$C_{18}H_{32}O_6$	-	tr	tr	tr	+	tr	[3]

	Compound name	RT	M-H (error in ppm)	MS^2	M+H (error in ppm)	MS^2	Formula	S1	S2	S3	S4	Am.	Chl.	Reference
23.	Oxo-octadecadienoic Acid isomer III	26.92			295.2267 (0.4)	277, 259, 249, 241, 179, 165, 151, 133, 125, 107, 95,	$C_{18}H_{30}O_3$	tr	+	-	+	+++	+	GNPS
24.	Dioxo-octadecadienoic acid	27.86	307.1915 (0.1)	289, 245, 209, 185, 165, 153, 113			$C_{18}H_{28}O_4$	-	+	+	+	+	+	[4]
25.	Oxohexadecanoic acid	33.33	269.2123 (-0.2)	225			$C_{16}H_{30}O_3$	tr	+	tr	+	+++	+	
	**Organic acid**													
26.	Phenylvalerate	16.99			197.1175 (-1.3)	179, 161, 151, 133	$C_{11}H_{14}O_2$	+	+	+	+	+	+	
	**Carotenoid**													
27.	Oxo-apo- caroten-one	17.74			289.1804 (-1.9)	271, 242, 229, 212, 200, 184	$C_{18}H_{24}O_3$	++	+	+	+++	-	+	[5]
	**Phospholipids**													
28.	Dioctanoyl phosphocholine	20.32			510.3189 (0.2)	258, 184, 104, 86	$C_{24}H_{49}NO_8P$	tr	tr	tr	tr	+	+++	
29.	Unknown glycerophosphocholine	20.91			536.3346 (0.2)	518, 500, 258, 184, 104	$C_{26}H_{51}NO_8P$	+	+	+	+	+	+	
30.	Unknown	21.16	523.2665 (2.4)	295, 277, 267, 227, 195, 152			$C_{24}H_{45}O_{10}P$	+	+	+	+	+	+	

	Compound name	RT	M-H (error in ppm)	MS^2	M+H (error in ppm)	MS^2	Formula	S1	S2	S3	S4	Am.	Chl.	Reference
31.	*O*-Ricinoleoyl- glycero-phosphocholine	22.33			538.3503 (0.0)	520, 502, 337, 258, 184, 104	$C_{26}H_{53}NO_8P^+$	tr	+	trt	tr	+++	+++	
32.	Unknown glycerophosphocholine	23.42			536.3344 (0.5)	184, 104	$C_{26}H_{51}NO_8P^+$	-	-	tr	tr	++	+++	
33.	*O*-Linoleoyl-*O*-stearoyl-sn-glycero-phosphate isomer I	31.67	699.4969 (0.2)	437, 279, 255			$C_{39}H_{73}O_8P$	-	-	-	+	+	+	GNPS, [6]
34.	*O*-Linoleoyl-*O*-stearoyl-sn-glycero-phosphate isomer II	31.98	699.497 (0.0)	437, 279, 255			$C_{39}H_{73}O_8P$	-	+	-	-	-	-	GNPS, [6]
35.	*O*-Linoleoyl-*O*-stearoyl-sn-glycero-phosphate isomer III	34.35	699.4966 (0.6)	437, 279, 255			$C_{39}H_{73}O_8P$	-	-	-	+	+	-	GNPS, [6]
	**Nitrogenous lipid**													
36.	Aminohexadecanediol	20.50			274.2741 (-0.1)	256, 230, 212, 133, 119, 106, 81, 70	$C_{16}H_{35}NO_2$	-	tr	+	+	++	+++	GNPS
37.	Aminotetradecanol	20.67			230.2478 (0.3)	212, 147, 135, 119, 105, 85, 81, 71	$C_{14}H_{31}NO$	-	-	-	tr	++	+++	
38.	Sphingenine, aminooctadec-enediol	20.74			300.2896 (0.3)	282, 272, 247, 191, 177, 163, 149, 135, 121, 105, 95, 81, 69	$C_{18}H_{37}NO_2$	-	-	-	tr	+++	tr	

	Compound name	RT	M-H (error in ppm)	MS²	M+H (error in ppm)	MS²	Formula	S1	S2	S3	S4	Am.	Chl.	Reference
39.	Aminoheptadecanediol	21.33			288.2900 (-1.0)	270, 134, 106, 88, 71	$C_{17}H_{37}NO_2$	-	tr	+++	-	-	tr	
40.	Amino-Icosanetetrol	23.26			362.3264 (0.2)	344, 314, 270, 238, 211	$C_{20}H_{43}NO_4$	-	-	-	-	+	+++	[7]
41.	Linoleic acid amide	23.74			296.2587 (-1.1)	278, 256, 209, 187, 161, 147, 135, 121, 109, 95, 81, 67	$C_{18}H_{33}NO_2$	-	tr	-	+	-	+++	[8]
42.	*N*-Palmitoyl-L-arginine	24.21			413.3489 (-0.7)	396, 354, 239, 175, 158	$C_{22}H_{44}N_4O_3$	-	++	tr	-	-	-	
43.	*N*-(dihydroxypropyl)octadecanamide	25.91			358.3315 (0.1)	340, 308, 298, 284, 266, 249, 217, 193, 165, 137, 123, 109, 95, 83, 69	$C_{20}H_{41}NO_2$	-	-	-	-	+++	tr	
44.	C20 sphingosine	26.27			328.3208 (0.6)	311, 282, 268, 237, 193, 135, 121, 109, 102, 95, 81, 69	$C_{20}H_{41}NO_2$	tr	-	-	tr	+++	+	
45.	C20 Sphinganine, icosasphinganine, dihydrosphingosine	27.31			330.3367 (-0.0)	312, 294, 282, 268, 217, 209, 193,133, 109, 95, 83, 67	$C_{20}H_{43}NO_2$	tr	-	-	-	+++	-	
46.	Aminoethyloleylamide	28.16			325.3208 (1.6)	307, 282, 268, 125, 109, 95, 81, 71	$C_{20}H_{40}N_2O$		-	-	+	+	+	

	Compound name	RT	M-H (error in ppm)	MS²	M+H (error in ppm)	MS²	Formula	S1	S2	S3	S4	Am.	Chl.	Reference
47.	Palmitoylputrescine	26.96			327.3368 (0.5)	309, 269, 235, 193, 181, 165, 121, 107, 95, 81, 69	$C_{20}H_{42}N_2O$	tr	tr	-		+++	+++	
48.	*N*-icosanoyl-glycine	29.77			370.3311 (1.3)	324, 102, 74	$C_{22}H_{43}NO_3$	-	-	tr	-	-	+++	
49.	Octadecanoyl-*L*-carnitine	30.02			428.3730 (1.1)	382, 364, 336, 207, 189, 144, 116, 98, 88	$C_{25}H_{49}NO_4$	-	-	-	+	++	+++	[9]
50.	Unknown fatty acyl amide	33.77			336.3264 (-0.9)	249, 235, 219, 205, 189, 175, 161, 147, 135, 123, 109., 95, 81, 69	$C_{22}H_{41}NO$	+	+	+	+++	+	+	
	**Phytoprostanes**													
51.	Phytoprostane A1	20.92			309.2057 (1.1)	291, 273, 263, 245, 227, 173, 145, 133, 119, 105, 93, 81	$C_{18}H_{28}O_4$	+	+	+	+	-	++	[10]
52.	Deoxyphytoprostane	22.13			291.196 (-2.0)	273, 245, 232, 193, 177, 161, 145, 135, 119, 105, 95, 81, 67	$C_{18}H_{26}O_3$	-	+	-	-	-	-	[11]
	**Glycolipid**													
53.	Hydroxydihydroprotolichesterinic acid-*O*-hexoside	22.50			555.3146 $[M+Na]^+$	393.2631, 259.079	$C_{27}H_{48}O_{10}$	+++	tr	++	++	tr	+	[12]

	Compound name	RT	M-H (error in ppm)	MS²	M+H (error in ppm)	MS²	Formula	S1	S2	S3	S4	Am.	Chl.	Reference
					(1.83)									
54.	Hydroxy-[(oxooctadecatrienyl)oxypropyl *O*-dihexoside	24.09	675.3582 (2.2)	415, 397, 277, 119			$C_{33}H_{56}O_{14}$	+	-	+++	+	-	tr	[13]
55.	*O*- Octadecaenoic-*O*-di-*O*-hexosyl-glycerol	25.41			701.3726 (1.73) [M+Na]+	539, 494, 409, 359, 347, 335, 313, 263, 245	$C_{33}H_{58}O_{14}$	+++	tr	++	++	tr	tr	
56.	Hexadecanoic acid-*O*-dihexosyl glycerol, isomer I	25.92	653.3748 (0.8)	415, 397, 255, 255, 89	655.3908 (-1.4)	331, 313, 257, 239, 163, 91	$C_{31}H_{58}O_{14}$	+	+	+	++	+	+	
57.	Hexadecanoic acid *O*-dihexosyl glycerol, isomer II	26.63	653.3752 (0.3)	415, 397, 255, 235, 161, 119, 89			$C_{31}H_{58}O_{14}$	+	+	+	+	+	+	
58.	*O*-linolenoyl-*O*-hexosyl-glycerol	27.17			537.3038 (1.18) $[M+Na]^+$	417, 375, 313, 299, 281, 259, 203	$C_{27}H_{46}O_9$	+++	tr	++	++	tr	tr	
59.	*O*-hexadecenoyl-hexosyl-glycerol	27.39			513.3039 (1.74) $[M+Na]^+$	261, 243, 203	$C_{25}H_{46}O_9$	++	-	++	+	tr	tr	
60.	(*O*-acetyl-hexosyl)oxy]octadecanoic acid	29.89			527.3198 (3.2)	313	$C_{26}H_{48}O_9$	+++	-	++	+	-	-	
61.	*O*-hexadecanoyl-hexosylglycerol	30.26			515.3197 (2.06)	353., 313, 259, 203, 185	$C_{25}H_{48}O_9$	++	+	++	+	tr	tr	

	Compound name	RT	M-H (error in ppm)	MS²	M+H (error in ppm)	MS²	Formula	S1	S2	S3	S4	Am.	Chl.	Reference
					[M+Na]⁺									
62.	*O*-palmitoyl-*O*-hexosylglycerol	30.91			493.3378 (-1.4)	331, 313, 257, 239, 221, 184, 137, 123, 109, 95, 85, 71	$C_{25}H_{48}O_9$	+	+	+	+	tr	+	[14]
63.	*O*-linoleoyl-*O*-palmitoyl-*O*-hexosyllglycerol	37.60			777.5502 (1.24) [M+Na]⁺	521, 497, 313.	$C_{43}H_{78}O_{10}$	-	-	+++	tr	tr	+	
	**Glycerolipids**													
64.	Monolinolenoyl glycerol	24.06			353.2688 (-0.5)	261, 243, 233, 217, 201, 187, 173, 163, 149, 135, 121, 107, 93, 81, 67	$C_{21}H_{36}O_4$	+	-	+	+	tr	tr	GNPS,
65.	Glyceryl palmitoleate	24.28			329.2694 (-2.3)	311, 299, 281, 257, 237, 219, 205, 163, 149, 135, 121, 109, 95, 83, 69	$C_{19}H_{36}O_4$	+++	tr	++	+	-	tr	GNPS
66.	Palmitoyl glycerol	26.52			331.2849 (-1.9)	313, 257, 239, 221, 151, 123, 109, 95, 85, 71	$C_{19}H_{38}O_4$	+	+	+	+	+	+	
67.	Linoleoyl glycerol	28.71			355.2847 (-1.2)	337, 263, 245, 203, 189, 175, 161, 147, 133, 123, 109, 95, 81, 67	$C_{21}H_{38}O_4$	+++	tr	+++	+++	-	tr	GNPS

	Compound name	RT	M-H (error in ppm)	MS²	M+H (error in ppm)	MS²	Formula	S1	S2	S3	S4	Am.	Chl.	Reference
68.	Linoleoyl Glycerol	29.24			355.2846 (-0.9)	337, 263, 245, 203, 189, 175, 161, 147, 133, 123, 109, 95, 81, 67	$C_{21}H_{38}O_4$	+++	tr	+++	+++	+++	tr	GNPS
	**Porphyrin**													
69.	Unknown	24.48			643.2771 (-1.3)	625, 593, 565, 335	$C_{35}H_{38}N_4O_8$	+	tr	+	+++	tr	+	
70.	Unknown	25.64			643.2770 (-1.2)	625, 593, 565, 335	$C_{35}H_{38}N_4O_8$	+	tr	+	+++	tr	+	
71.	Unknown	28.78			657.2925 (-0.9)	639, 607, 579, 487, 349, 333, 313	$C_{36}H_{40}$N4O $_8$	++	tr	++	++	tr	+	
72.	Unknown	29.92			627.2454 (-0.8)	609, 595, 577, 549	$C_{34}H_{34}N_4O_8$	+	tr	+	+++	tr	tr	
73.	Unknown	30.30			625.2664 (-1.1)	607, 575, 547, 531, 501	$C_{35}H_{36}N_4O_7$	+	+	+	+++	tr	+	[15]
74.	Bacteriopheophorbide a	31.14			611.2869 (-0.8)	567, 538, 494, 465, 317	$C_{35}H_{38}N_4O$ 6	+	-	+	+++	-	+	[16]
75.	Unknown	31.19			583.2555 (-0.7)	565, 539, 521, 503, 495, 477, 466	$C_{33}H_{34}N_4O_6$	+	+	+	+++	+	+	
76.	Unknown	33.88			569.2766 (-1.3)	551, 536., 519, 482, 464, 449, 325, 291	$C_{33}H_{36}N_4O_5$	+	-	+	+++	-	+	

	Compound name	RT	M-H (error in ppm)	MS^2	M+H (error in ppm)	MS^2	Formula	S1	S2	S3	S4	Am.	Chl.	Reference
77.	Mesoporphyrin IX	34.09			567.2974 (-1.5)	535, 507, 494, 479, 421, 291	$C_{34}H_{38}N_4O_4$	-	-	-	+++	-	tr	
78.	Unknown	34.26			597.2715 (-1.3)	579, 565, 553, 537, 521, 503, 477	$C_{34}H_{36}N_4O_6$	+	+	+	+++	+	++	
79.	Unkown	36.19			625.3026 (-0.9)	565., 552, 479, 313	$C_{36}H_{40}N_4O_6$	+	tr	+	+++	-	tr	
80.	Harderoporphyrin<br>Ethenyl-3,8,13,17-tetramethyl-21*H*,23*H*-porphine-2,7,18-tripropanoic acid	36.92			609.2720 (-2.0)	591, 577, 559, 549, 531, 503, 485	$C_{35}H_{36}N_4O_6$	+++	tr	++	++	tr	tr	[17]
81.	Methyl 8-ethyl-12-methylbacteriopheophorbide c	37.24			581.3132 (-1.7)	508, 421, 300	$\underline{C_{35}H_{40}N_4O_4}$	-	-		+++	-	-	
82.	Tetramethyl-3-(methoxycarbonyl)-5-(1,2-dioxo-2-methoxyethyl)-13-vinyl-18-ethyl-7,8-dihydro-21H,23H-porphyrin-7-propionic acid	37.97			639.2823 (-1.5)	607, 579, 552, 537, 493, 479	$\underline{C_{36}H_{38}N_4O_7}$	+++	+	+	++	tr	+	[18]
83.	Hydroxy pheophorbide a	39.35			609.2707 (0.1)	550, 309, 101	$C_{35}H_{36}N_4O_6$	+	+	+	+++	tr	tr	[19]

	Compound name	RT	M-H (error in ppm)	MS²	M+H (error in ppm)	MS²	Formula	S1	S2	S3	S4	Am.	Chl.	Reference
84.	Pheophorbide a	39.69			593.2753 (1.0)	533, 459	$C_{35}H_{36}N_4O_5$	-	+	-	+	-	-	[20]
	**Hydrocarbons**													
85.	Octadecatetraenal	27.17			261.2215 (-0.7)	243, 233, 217, 201, 187, 177, 161, 145, 131, 121, 107, 93, 81, 67	$C_{18}H_{28}O$	+++	-	+++	++	tr	+	
	**Sulpholipids**													
86.	*O*-palmitoyl-*O*-(sulfo-galactosyl) glycerol isomer I	31.82	555.2848 (-0.5)	225			$C_{25}H_{48}O_{11}S$	-	+++	+	-	-	-	[21]
87.	*O*-palmitoyl-*O*-(sulfo-hexosyl) glycerol isomer II	32.23	555.2848 (-0.6)	299, 255, 225			$C_{25}H_{48}O_{11}S$	+++	-	-	+	-	-	[21]
	**Carboximidic acid**													
88.	*N*-(hydroxyethyl)-hexadecenamide	32.42			298.2736 (1.6)	279, 251, 233, 219, 183, 153, 135, 121, 107, 95, 83, 69	$C_{18}H_{35}NO_2$	+	+	+++	-	-	++	
89.	*N*-(hydroxyethyl) octadecadienamide	33.43			324.2900 (-0.9)	306, 284, 147, 121, 95, 81,62	$C_{20}H_{37}NO_2$	+	+	+++	+++	++	++	GNPS

Supplementary Table S2: The relative percentage of silylated metabolites in *Spirulina* (*S1, S2 & S3*), *Amphora* (Am.) and *Chlorella* (Chl.) samples analyzed via GC-MS, n= 3

Compound number	Average Rt(min)	Average RI	Metabolite name	Chemical Class	S1	S3	S4	Am.	Chl.
	**Acohols**								
1.	6.413	1141.34	2,3-Butanediol, 2TMS	Alcohol	0.28 ±0.07	0.09 ±0.01	0.07 ±0.001	0.56 ±0.12	0.36 ±0.07
2.	6.943	1174.21	Glycerol, 2TMS	Alcohol	1.11 ±0.14	0.72 ±0.09	0.46 ±0.02	0.20 ±0.04	0.99 ±0.47
3.	8.321	1269.18	Glycerol, 3TMS	Alcohol	0.14 ±0.01	0.49 ±0.18	0.10 ±0.01	5.28 ±0.76	3.54 ±0.68
	**Total Alcohols**				**1.57**	**1.31**	**0.64**	**6.04**	**4.89**
	**Amino acids**								
4.	5.805	1103.54	Alanine, 2TMS	Amino acid	0.88 ±0.06	1.96 ±0.72	0.65 ±0.09	12.33 ±1.89	3.26 ±0.58
5.	7.18	1189.07	Valine, 2TMS	Amino acid	1.42 ±0.10	1.52 ±0.30	0.87 ±0.02	0.24 ±0.09	1.14 ±0.35
6.	7.51	1210.96	Leucine, 2TMS	Amino acid	0.22 ±0.01	0.42 ±0.13	0.15 ±0.01	7.30 ±1.05	4.84 ±0.79
7.	7.76	1228.92	Isoleucine, 2TMS	Amino acid	0.69 ±0.23	0.27 ±0.05	0.18 ±0.04	1.03 ±0.19	2.72 ±0.51
8.	8.633	1291.56	Allo-isoleucine, 2TMS	Amino acid	0.13 ±0.01	0.22 ±0.06	0.08 ±0.001	3.31 ±0.46	2.24 ±0.39
9.	9.965	1387.15	Serine, 3TMS	Amino acid	1.03 ±0.10	0.19 ±0.09	0.12 ±0.02	0.48 ±0.05	0.68 ±0.30
10.	11.057	1473.52	Aspartic acid, 3TMS	Amino acid	0.08 ±0.01	0.16 ±0.02	0.10 ±0.02	0.82 ±0.10	0.55 ±0.12
11.	11.635	1520.16	L-5-Oxoproline, 2TMS	Amino acid	0.68 ±0.18	1.13 ±0.45	0.12 ±0.01	1.09 ±0.16	0.16 ±0.07
12.	12.882	1622.94	Glutamic acid, 3TMS	Amino acid	1.23 ±0.64	0.30 ±0.13	0.09 ±0.03	1.09 ±0.65	1.65 ±0.32
13.	12.893	1624	Phenylalanine, 2TMS	Amino acid	0.01 ±0.001	0.09 ±0.04	0.01 ±0.001	0.73 ±0.07	0.35 ±0.09
14.	16.252	1939	Tyrosine, 3TMS	Nitrogenous compound	0.04 ±0.01	0.09 ±0.02	0.02 ±0.001	0.30 ±0.02	0.62 ±0.14
	**Total Amino Acids**				**6.41**	**6.36**	**2.39**	**28.71**	**18.12**
	**Fatty acids/ esters**								
15.	14.936	1808.39	Myristic acid, TMS	Fatty acid/ester	0.33 ±0.09	0.13 ±0.02	0.33 ±0.19	0.30 ±0.02	0.96 ±0.39
16.	15.939	1908.4	Palmitic acid, methyl ester	Fatty acid/ester	0.72 ±0.11	0.26 ±0.03	0.24 ±0.03	0.56 ±0.06	1.28 ±0.39
17.	17.116	2027.1	Palmitic Acid, TMS	Fatty acid/ester	20.22 ±0.58	15.23 ±3.73	9.24 ±0.16	10.18 ±1.51	8.31 ±2.08
18.	18.483	2175.67	Linolenic acid, TMS	Fatty acid/ester	0.63 ±0.08	0.76 ±0.09	0.56 ±0.03	0.35 ±0.10	0.20 ±0.09
19.	18.638	2192.52	Linoleic acid isomer, TMS	Fatty acid/ester	1.76 ±0.03	2.40 ±0.27	1.74 ±0.07	1.85 ±0.17	1.38 ±0.57

20.	18.682	2197.28	Oleic Acid, TMS	Fatty acid/ester	0.19 ±0.02	0.34 ±0.03	0.20 ±0.03	0.22 ±0.03	0.16 ±0.06
21.	18.895	2222.22	Stearic acid, TMS	Fatty acid/ester	6.86 ±0.46	6.04 ±2.64	0.16 ±0.02	0.73 ±0.15	0.86 ±0.12
22.	21.482	2538.3	Arachidic acid, TMS	Fatty acid/ester	0.31 ±0.05	0.21 ±0.001	0.23 ±0.08	0.41 ±0.03	1.05 ±0.24
23.	21.499	2540.5	2-Palmitoylglycerol, 2TMS	Fatty acid/ester	0.09 ±0.03	0.05 ±0.01	0.02 ±0.01	0.07 ±0.06	0.14 ±0.08
24.	21.757	2573.46	1-Monopalmitin, 2TMS	Fatty acid/ester	3.36 ±1.43	2.05 ±0.84	0.53 ±0.29	7.58 ±3.65	8.94 ±6.26
25.	23.164	2764.98	Glycerol monostearate, 2TMS	Fatty acid/ester	3.44 ±1.59	2.15 ±1.06	0.48 ±0.34	9.35 ±4.83	9.65 ±7.66
	**Total Fatty Acid/ Esters**				**37.93**	**29.63**	**13.72**	**31.60**	**32.93**
	**Glycosides**								
26.	19.365	2277.58	Glyceryl-glycoside TMS ether	Glycoside	22.68 ±3.14	27.38 ±12.28	40.11 ±0.17	Traces	0.36 ±0.16
27.	19.769	2325.14	2-*O*-Glycerol-galactopyranoside, 6TMS	Glycoside	1.03 ±0.03	1.75 ±0.07	0.36 ±0.15	0.15 ±0.07	2.74 ±0.94
28.	20.374	2396.6	2-*O*-Glycerol-galactopyranoside, 6-TMS	Glycoside	0.51 ±0.11	0.35 ±0.03	0.38 ±0.11	0.57 ±0.09	0.82 ±0.29
	**Total Glycosides**				**24.22**	**29.48**	**40.85**	**0.72**	**3.92**
	**Hydrocarbons**								
29.	13.563	1684.17	Pentadecane, 2,6,10-trimethyl-	Hydrocarbon	1.23 ±0.11	0.51 ±0.14	0.17 ±0.01	0.03 ±0.01	0.07 ±0.02
	**Total Hydrocarbons**				**1.23**	**0.51**	**0.17**	**0.03**	**0.07**
	**Inorganic compounds**								
30.	14.57	1774.68	Phosphoric acid, 2,3-(2-TMS)propyl (2-TMS) ester	Inorganic Compound	0.13 ±0.03	0.04 ±0.01	0.03 ±0.01	1.09 ±0.20	0.46 ±0.14
31.	18.718	2201.34	Phosphoric acid, 2[2,3-(2-TMS)oxy]propyl] TMS, ester	Inorganic Compound	0.05 ±0.001	0.04 ±0.01	0.02 ±0.001	0.07 ±0.02	0.26 ±0.03
	**Total Inorganic Compounds**				**0.18**	**0.08**	**0.05**	**1.16**	**0.72**
	**Nitrogenous compounds**								
32.	6.921	1172.94	2-Aminobutanoic acid	Nitrogenous compound	0.50 ±0.14	0.16 ±0.03	0.13 ±0.03	0.36 ±0.05	0.42 ±0.09
33.	8.379	1273.49	Niacin, TMS	Nitrogenous compound	2.62 ±0.09	3.66 ±1.09	5.99 ±0.04	11.98 ±1.46	9.27 ±2.03
34.	9.031	1320.13	Unknown nitrogenous compound	Nitrogenous compound	0.23 ±0.06	0.12 ±0.001	0.09 ±0.001	0.21 ±0.05	0.08 ±0.04
35.	9.584	1359.79	Uracil, 2TMS	Nitrogenous compound	0.17 ±0.04	0.22 ±0.06	0.03 ±0.01	0.09 ±0.01	0.04 ±0.01

36.	15.176	1832.51	Adenine, 2TMS	Nitrogenous compound	0.59 ±0.09	0.25 ±0.04	0.16 ±0.03	0.96 ±0.11	2.04 ±0.26
37.	22.265	2641.37	Adenosine, 4TMS	Nitrogenous compound	0.27 ±0.10	0.24 ±0.03	0.18 ±0.07	0.95 ±0.29	0.44 ±0.16
	**Total Nitrogenous Compounds**				**4.38**	**4.65**	**6.59**	**14.56**	**12.31**
	**Organic acids**								
38.	8.857	1307.63	Succinic acid, 2-TMS	Oragnic acid	1.64 ±0.07	1.30 ±0.17	1.32 ±0.05	2.18 ±0.27	0.48 ±0.12
39.	5.094	1059.37	Lactic Acid, 2TMS	Organic acid	0.26 ±0.07	0.07 ±0.001	0.07 ±0.01	1.24 ±0.30	0.5 ±0.09
40.	5.175	1064.38	Caproic acid, TMS	Organic acid	5.80 ±0.70	5.39 ±0.66	3.65 ±0.22	7.95 ±1.16	5.77 ±1.16
41.	5.385	1077.44	Glycolic acid, 2TMS	Organic acid	0.58 ±0.08	0.39 ±0.09	0.19 ±0.01	0.32 ±0.03	0.48 ±0.15
42.	5.566	1088.72	Pyruvic acid, 2-TMS	Organic acid	0.45 ±0.08	0.12 ±0.01	0.07 ±0.01	0.53 ±0.06	1.76 ±0.86
43.	6.215	1129.06	Methylmalonic acid, 2TMS	Organic acid	0.60 ±0.17	0.18 ±0.02	0.14 ±0.02	0.68 ±0.10	1.14 ±0.25
44.	6.662	1156.79	3-Hydroxybutyric acid, 2TMS derivative	Organic acid	1.70 ±0.16	3.01 ±0.70	1.59 ±0.02	0.64 ±0.10	0.56 ±0.17
45.	9.18	1330.8	Glyceric acid, 3TMS	Organic acid	0.26 ±0.03	0.38 ±0.06	0.25 ±0.01	0.20 ±0.03	0.29 ±0.05
46.	10.523	1430.54	3,4-dihydroxybutyric acid, 3-TMS	Organic acid	0.31 ±0.06	0.21 ±0.05	0.10 ±0.01	0.34 ±0.10	0.31 ±0.08
	**Total Organic Acids**				**11.60**	**11.06**	**7.38**	**14.08**	**11.28**
	**Phenolics**								
47.	12.808	1616.29	1,2,4-Benzenetriol, 3-TMS ether	Phenolic	2.79 ±0.23	0.90 ±0.04	0.70 ±0.03	0.20 ±0.02	Traces
	**Total Phenolics**				**2.79**	**0.90**	**0.70**	**0.20**	**Traces**
	**Sugars**								
48.	15.594	1873.69	Rhamnose, 4TMS	Sugar	0.15 ±0.02	0.10 ±0.01	0.05 ±0.01	0.32 ±0.09	0.50 ±0.07
49.	15.924	1906.45	Glucose, 5TMS derivative	Sugar	0.09 ±0.03	0.08 ±0.03	0.03 ±0.01	0.09 ±0.02	0.78 ±0.52
50.	16.481	1961.72	Glucose isomer, 5TMS derivative	Sugar	0.20 ±0.05	0.13 ±0.02	0.31 ±0.14	0.27 ±0.05	0.90 ±0.50
51.	19.844	2334.16	3-Mannobiose, 8-TMS ether isomer	Sugar	7.42 ±0.83	13.37 ±0.77	24.76 ±0.10	0.18 ±0.04	2.74 ±0.94
52.	20.195	2375.54	Maltose, 8TMS derivative	Sugar	0.16 ±0.01	0.24 ±0.02	0.58 ±0.02	0.03 ±0.03	Traces
53.	22.524	2677	Sucrose, 8TMS	Sugar	0.63 ±0.19	0.67 ±0.42	0.23 ±0.09	0.90 ±0.20	8.56 ±4.68
54.	26.331	3072.06	Melibiose isomer, 8TMS	Sugar	0.42 ±0.03	0.91 ±0.33	1.04 ±0.02	0.46 ±0.11	1.01 ±0.37
	**Total Sugars**				**9.07**	**15.49**	**27.00**	**2.25**	**14.48**

55.	17.86	2107.63	Myo-Inositol, 6TMS	Sugar alcohol	0.06 ±0.01	0.06 ±0.01	0.03 ±0.001	0.15 ±0.01	0.10 ±0.06
	**Total Sugar Alcohols**				**0.06**	**0.06**	**0.03**	**0.15**	**0.10**
	**Terpene alcohols**								
56.	18.326	2158.52	Phytol, TMS	Terpene alcohol	0.57 ±0.10	0.48 ±0.06	0.48 ±0.01	0.51 ±0.05	1.08 ±0.34
	**Total Terpene Alcohols**				**0.57**	**0.48**	**0.48**	**0.51**	**1.08**

Supplementary Table S3: Sample codes

Sample code	Sample	Source
S1	Spirulina	spring valley, USA
S2	Spirulina tablets	GNC superfoods, USA
S3	Spirulina tablets	Gse vertrieb Gmbh, Germany
S4	Spirulina powder	Imtenan, Egypt
Am.	Amphora powder	NRC
Chl.	Chlorella powder	NRC

Supplementary Table S4: Parameters used for the construction of the feature-based molecular networks

Precursor ion mass tolerance	0.05
Fragment ion mass tolerance	0.05
Cosine score	0.7
Minimum matching fragments	6

Supp. Fig. S1: Base Peak Chromatogram (BPC) of the studied algal species in the negative ionization mode.

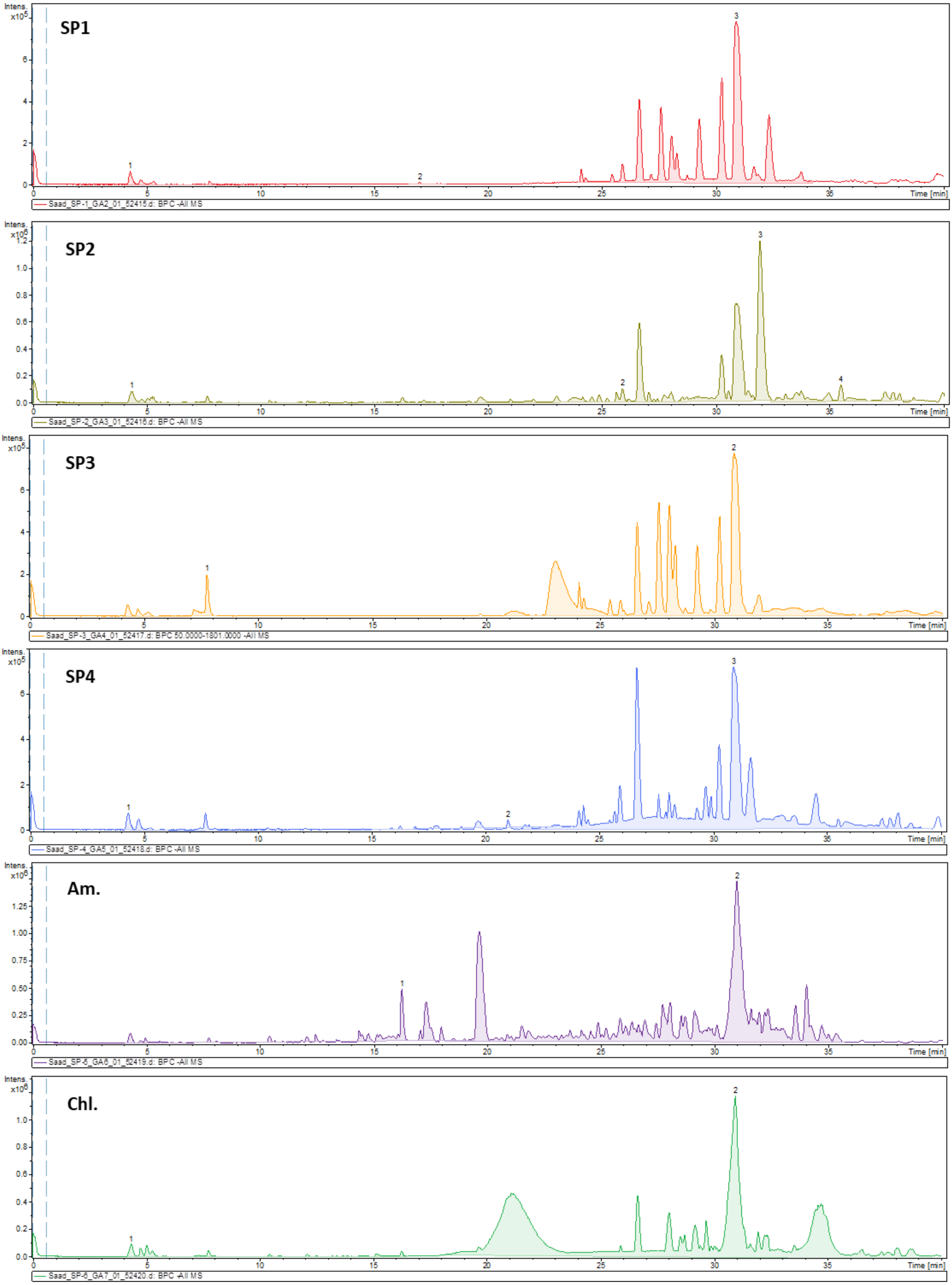

Supp. Fig. S2: Feature-based Molecular Network (FBMN) of acquired UPLC-HRMS/MS data (negative ionization mode) of Spirulina samples versus Amphora and Chlorella. Clusters a-g: fatty acids, h: phospholipids, i: glycolipids, and j: sulpholipids.

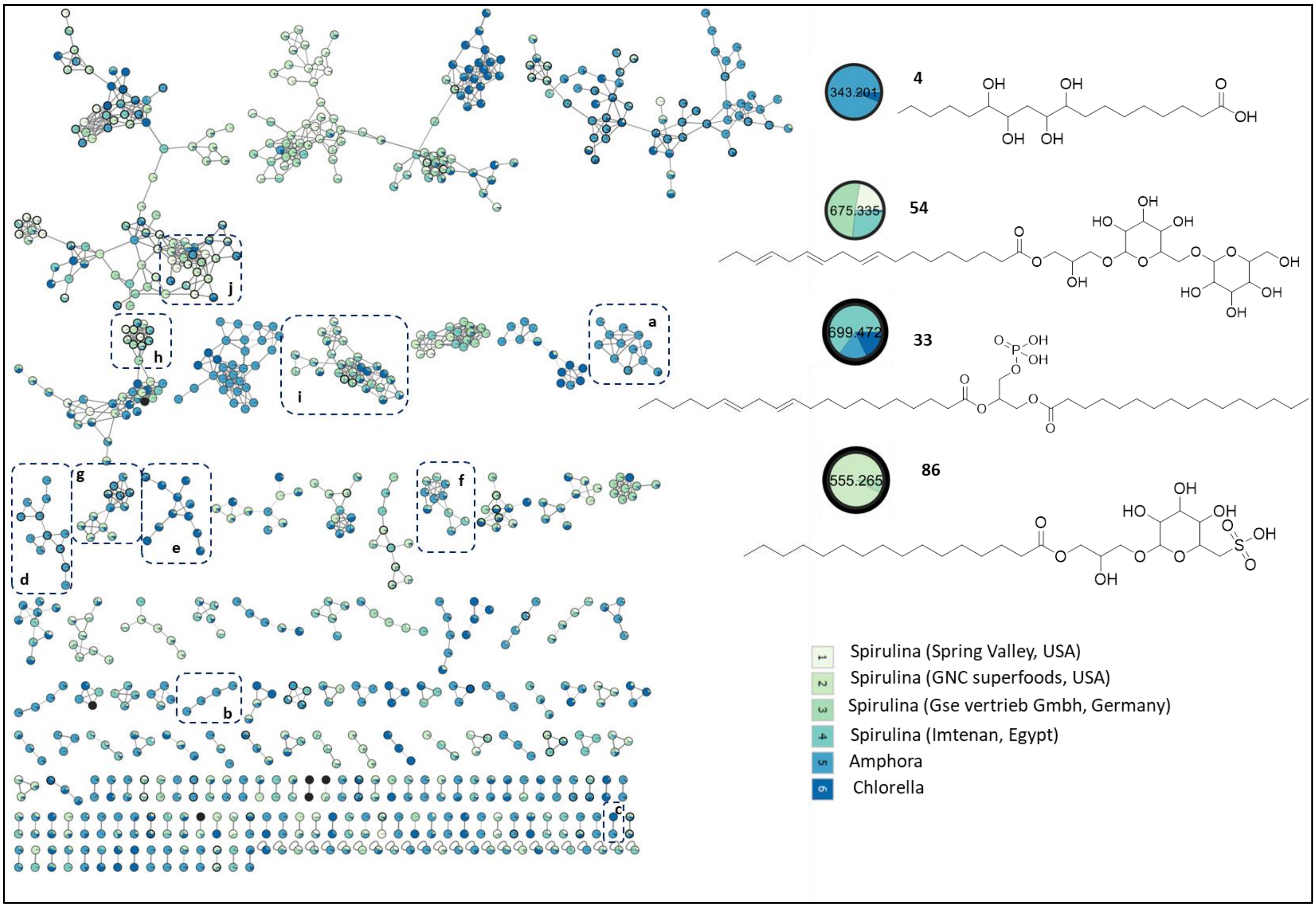

Supp. Fig. S3: MS^2 spectrum of compound 4, tetrahydroxy octadecadienoic acid in the negative ionization mode

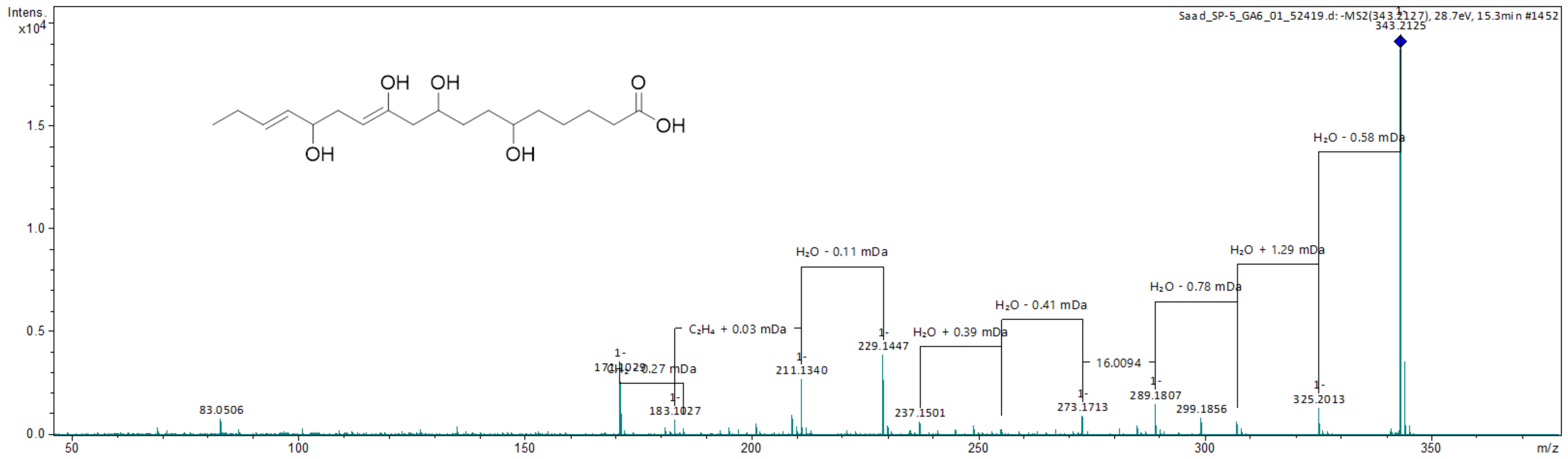

Supp. Fig. S4: MS^2 spectrum of compound 8, oxo-octadecadienoic acid in the positive ionization mode

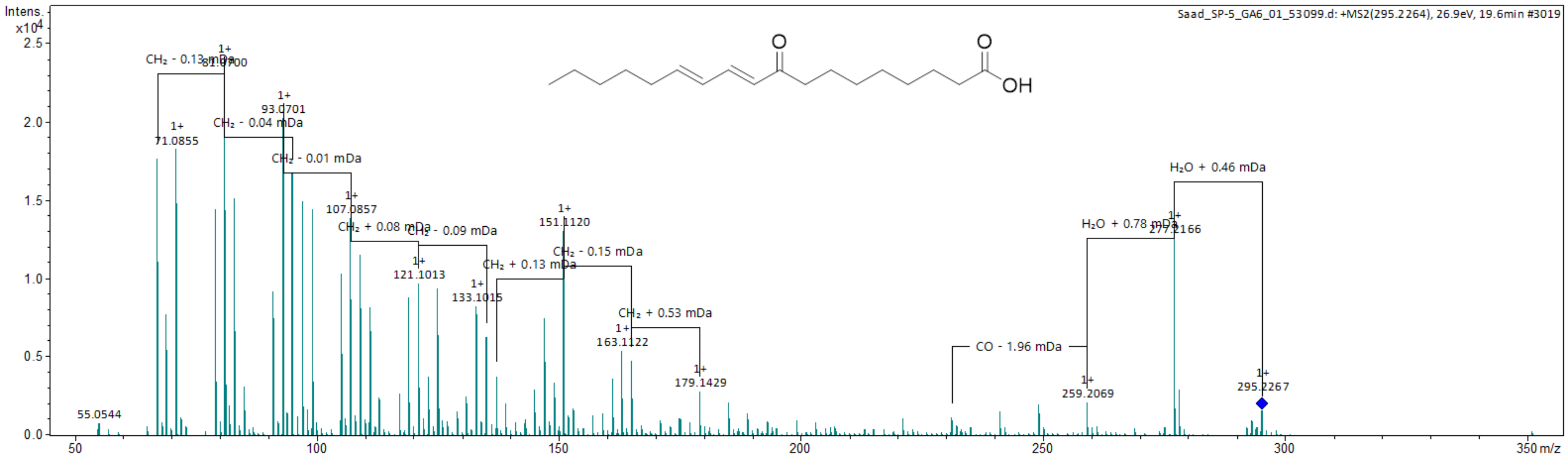

Supp. Fig. S5: MS^2 spectrum of compound 28, dioctanoyl phosphocholine in the positive ionization mode

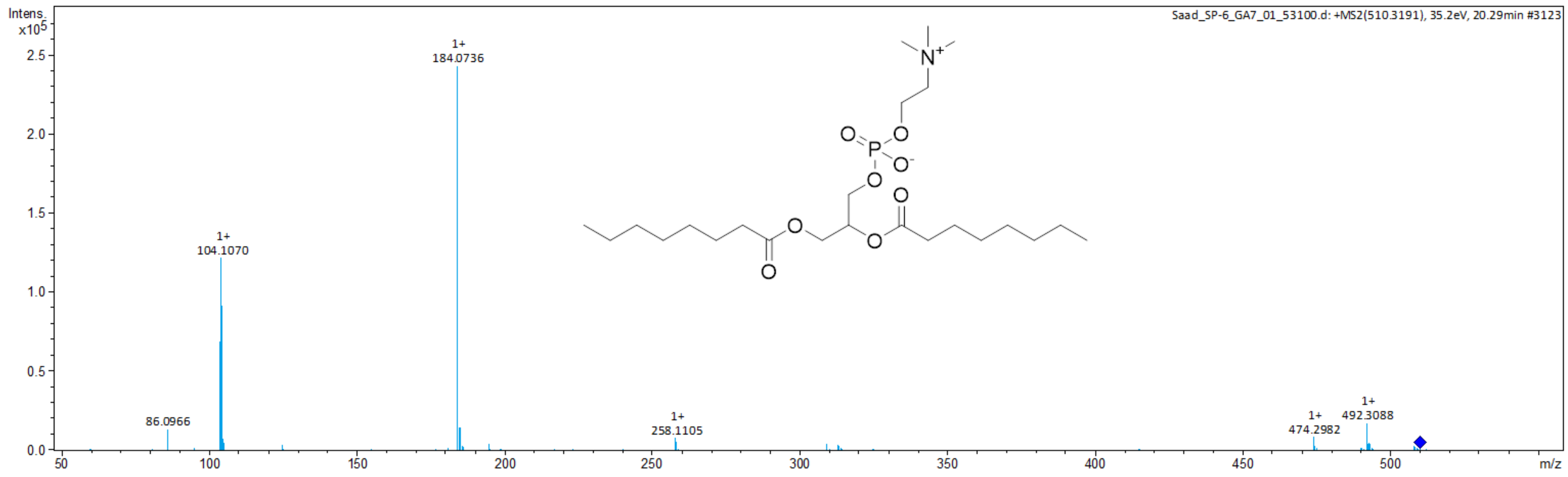

Supp. Fig. S6: MS^2 spectrum of compound 33, of hexadecanoyl-2-(eicosadienoyl)-glycerophosphate in the positive ionization mode

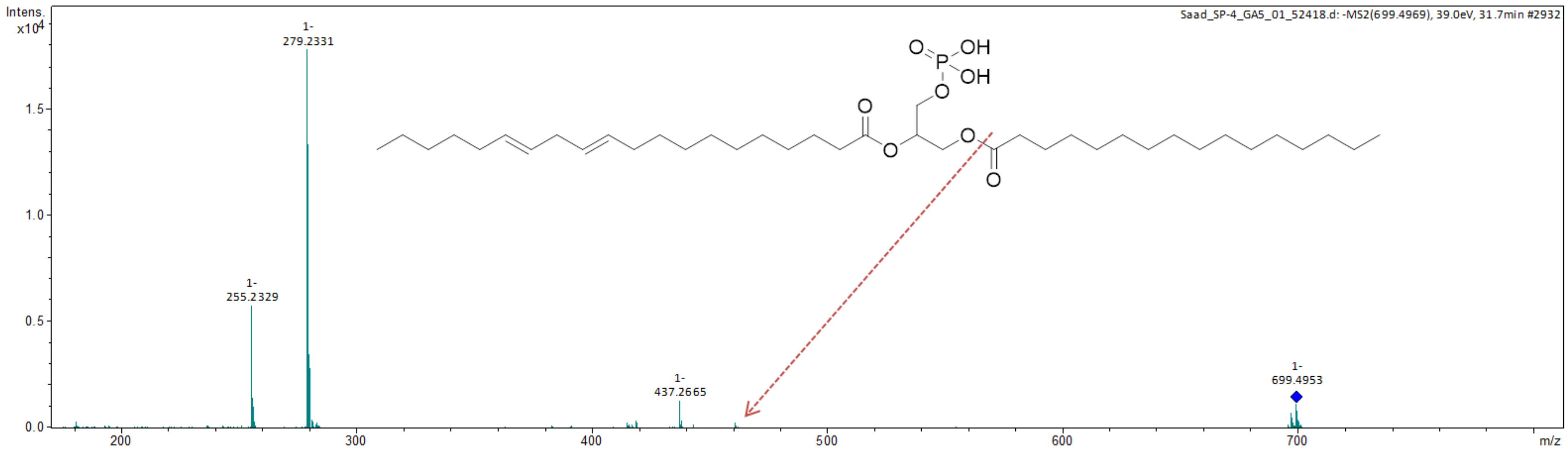

Supp. Fig. S7: MS^2 spectrum of compound 38, of aminooctadecenediol in the positive ionization mode

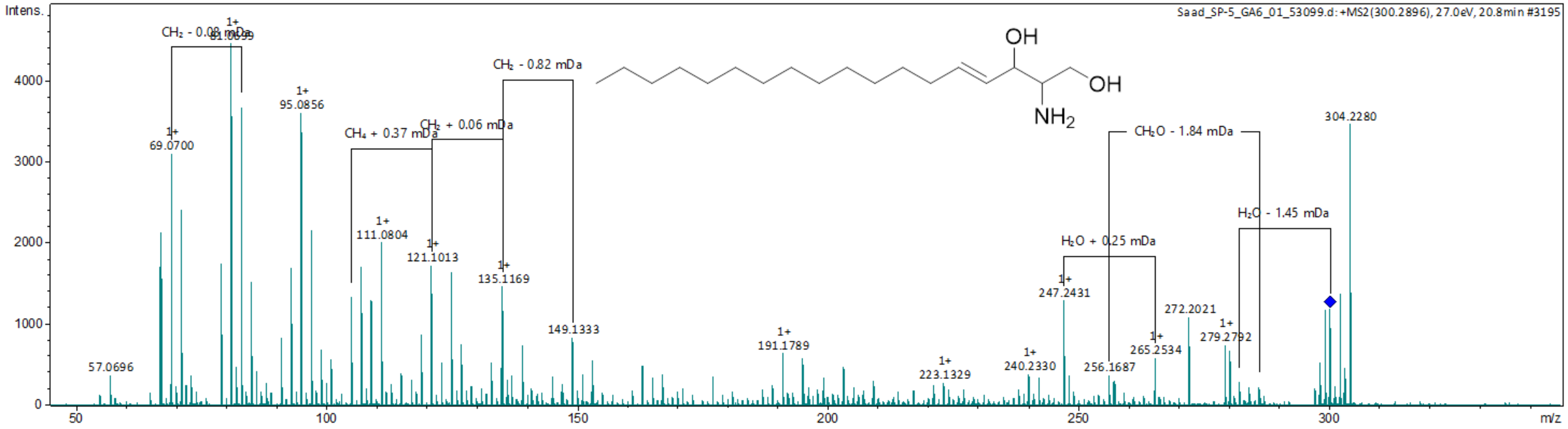

Supp. Fig. S8: MS^2 spectrum of compound 41, of linoleic acid amide in the positive ionization mode

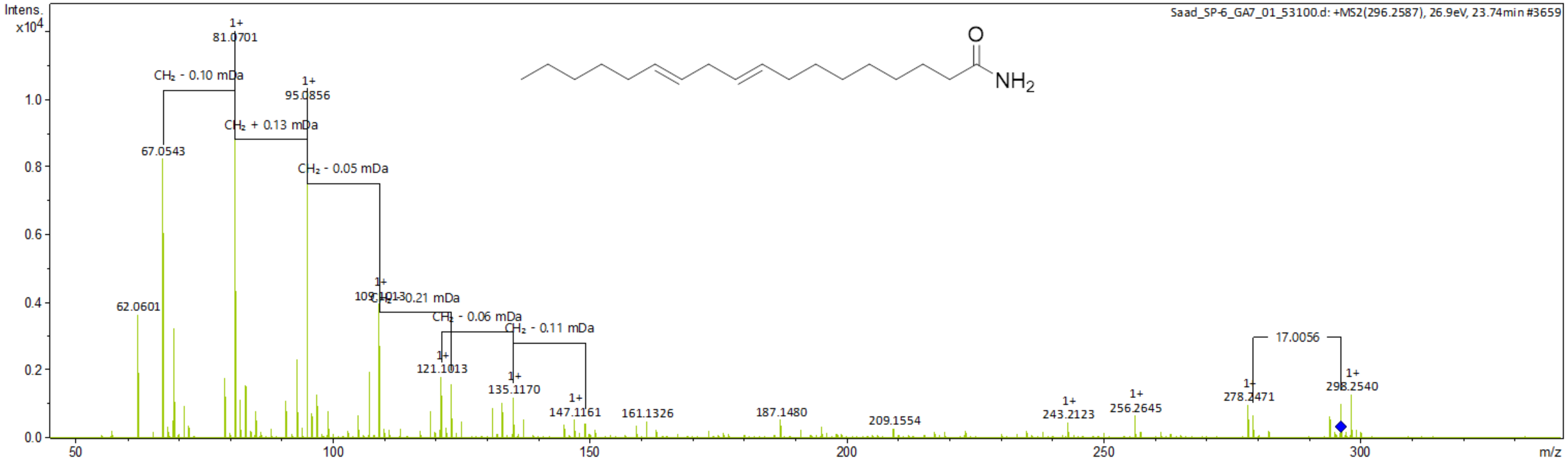

Supp. Fig. S9: MS^2 spectrum of compound 42, of *N*-palmitoyl-L-arginine in the positive ionization mode

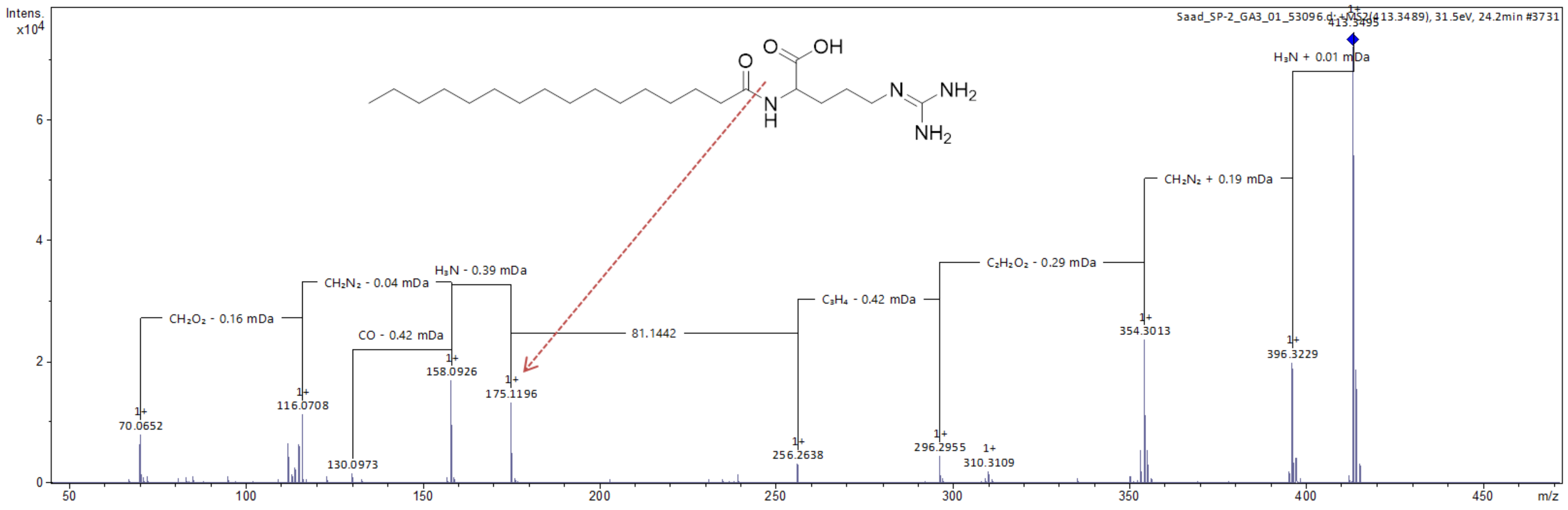

Supp. Fig. S10: MS^2 spectrum of compound 52, of Hexadecanoic acid-*O*-dihexosyl glycerol in the positive ionization mode

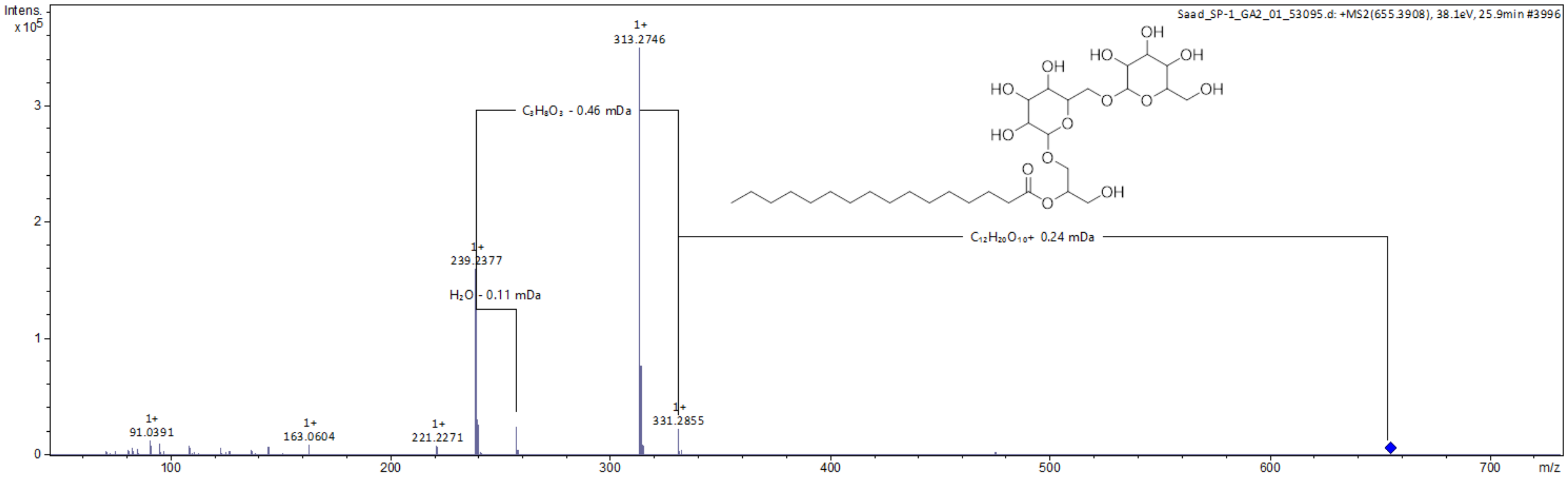

Supp. Fig. S11: MS[2] spectrum of compound 58, of *O-palmitoyl-O- hexosylglycerol* in the positive ionization mode

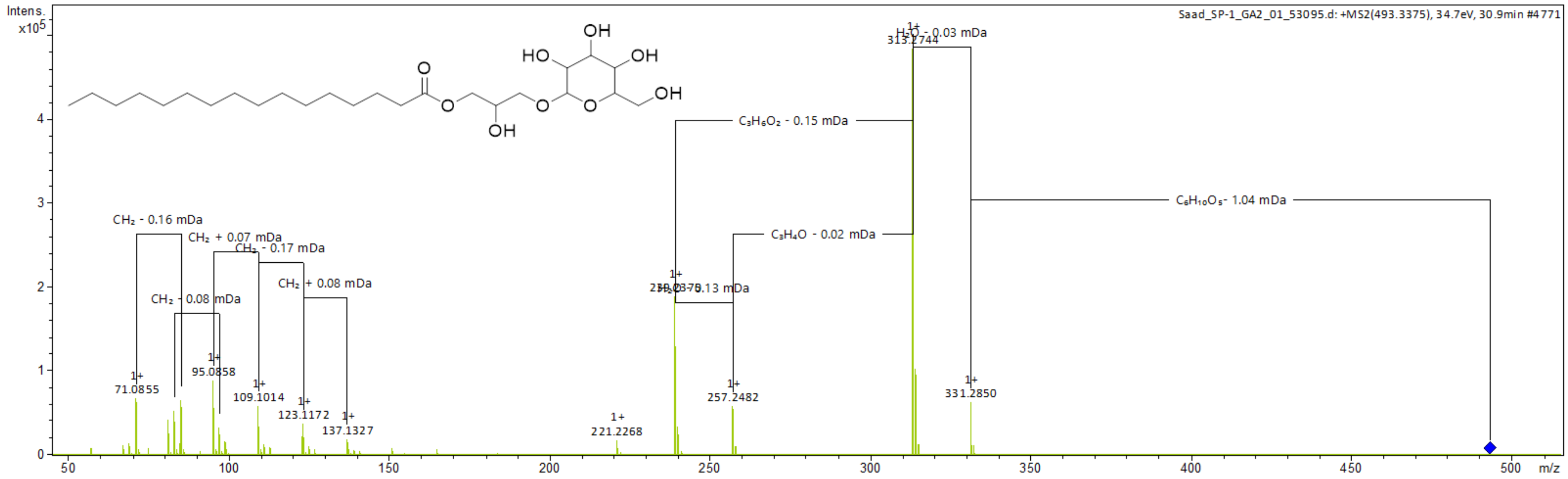

Supp. Fig. S12: MS[2] spectrumof compound 62, of *O-palmitoylglycerol* in the positive ionization mode

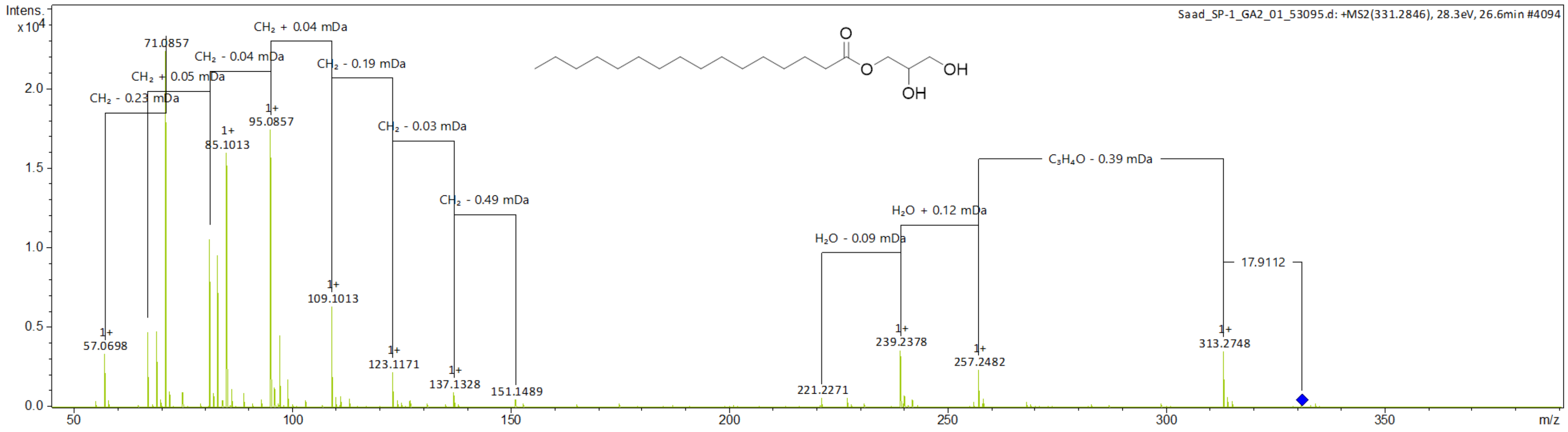

Supp. Fig. S13: MS² spectrum of compound 87, of *O-palmitoyl-O-(sulfo- hexosyl) glycerol* in the negative ionization mode

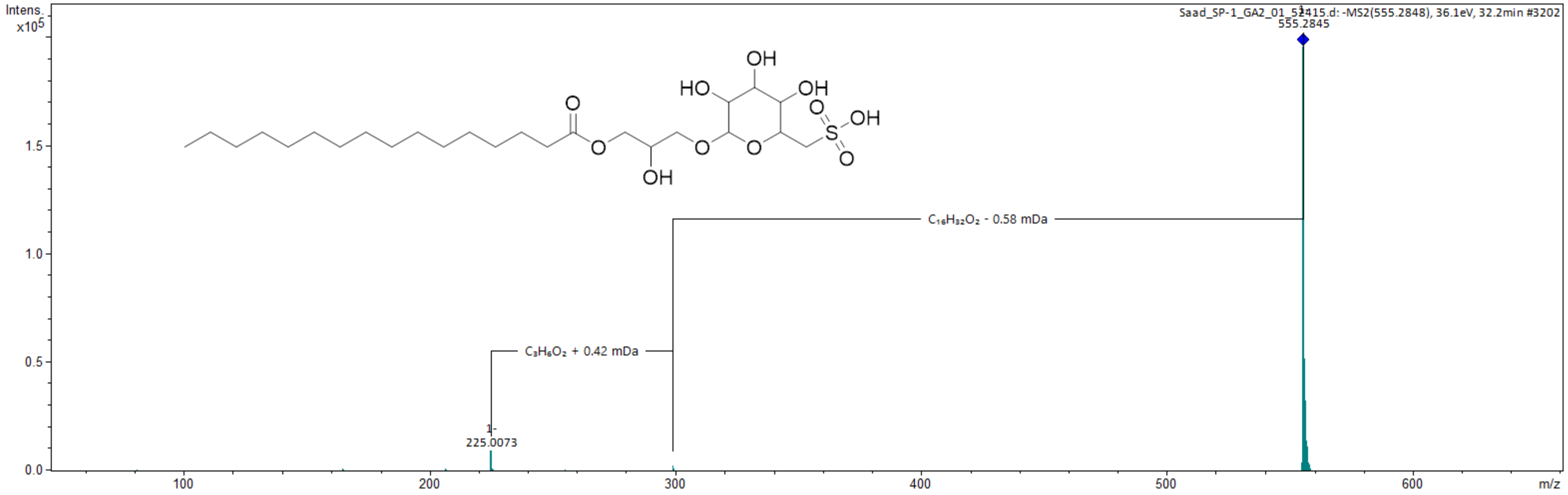

Supp. Fig. S14: UV and MS^2 spectra of compound 69, of 10-Ethenyl-5-ethyl-1,15,16,18-tetrahydro-18-hydroxy-18-(methoxycarbonyl)-6,11,15,22-tetramethyl-20-oxo-20H-9,12-imino-2,21-metheno-7,4:14,17-dinitrilopyrano[4,3-b]azacyclononadecine-16-propanoic acid in the positive ionization mode

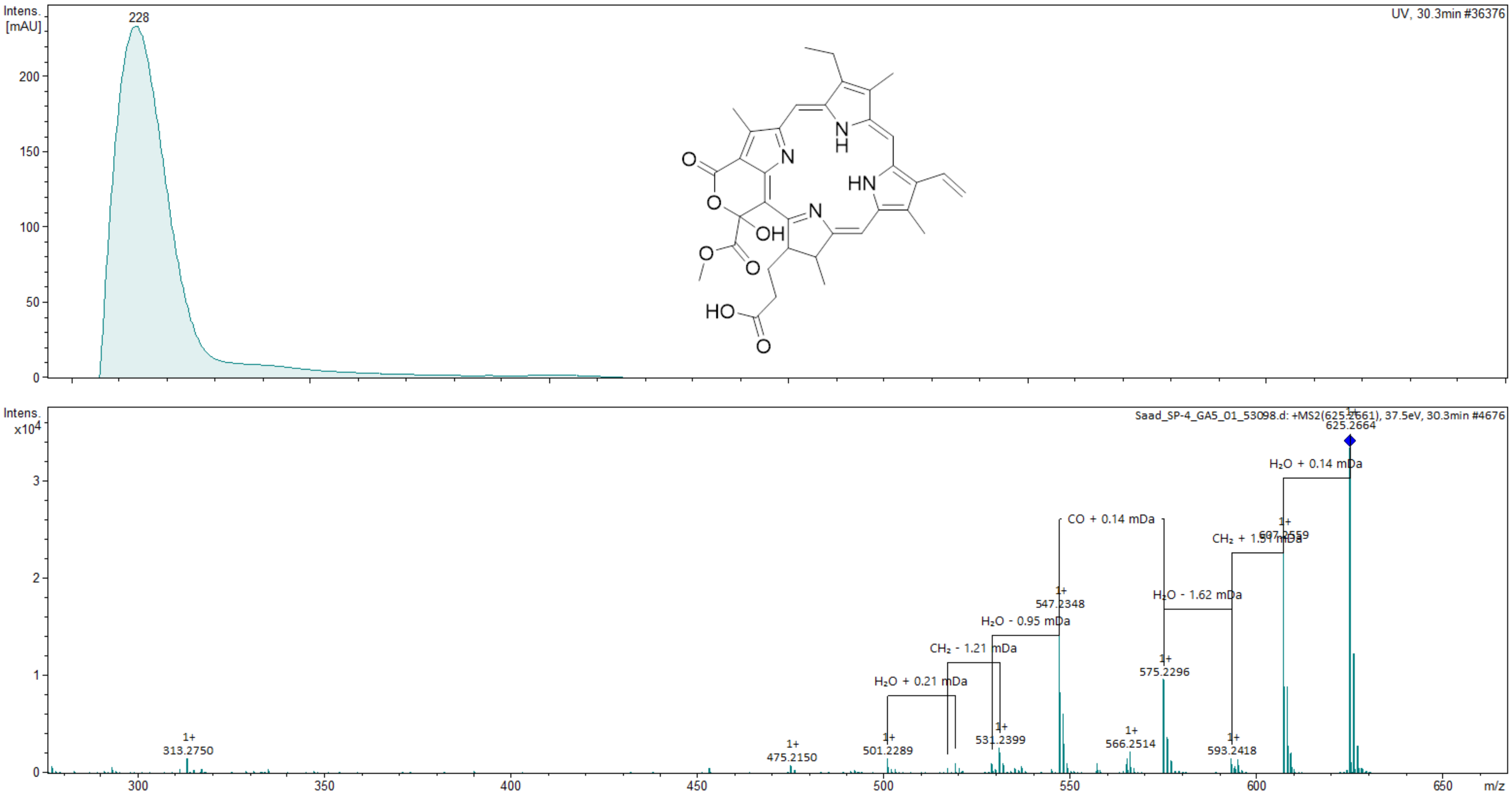

Supp. Fig. S15: UV and MS² spectra of compound MS² spectra of compound 76, of harderoporphyrin isomer I in the positive ionization mode

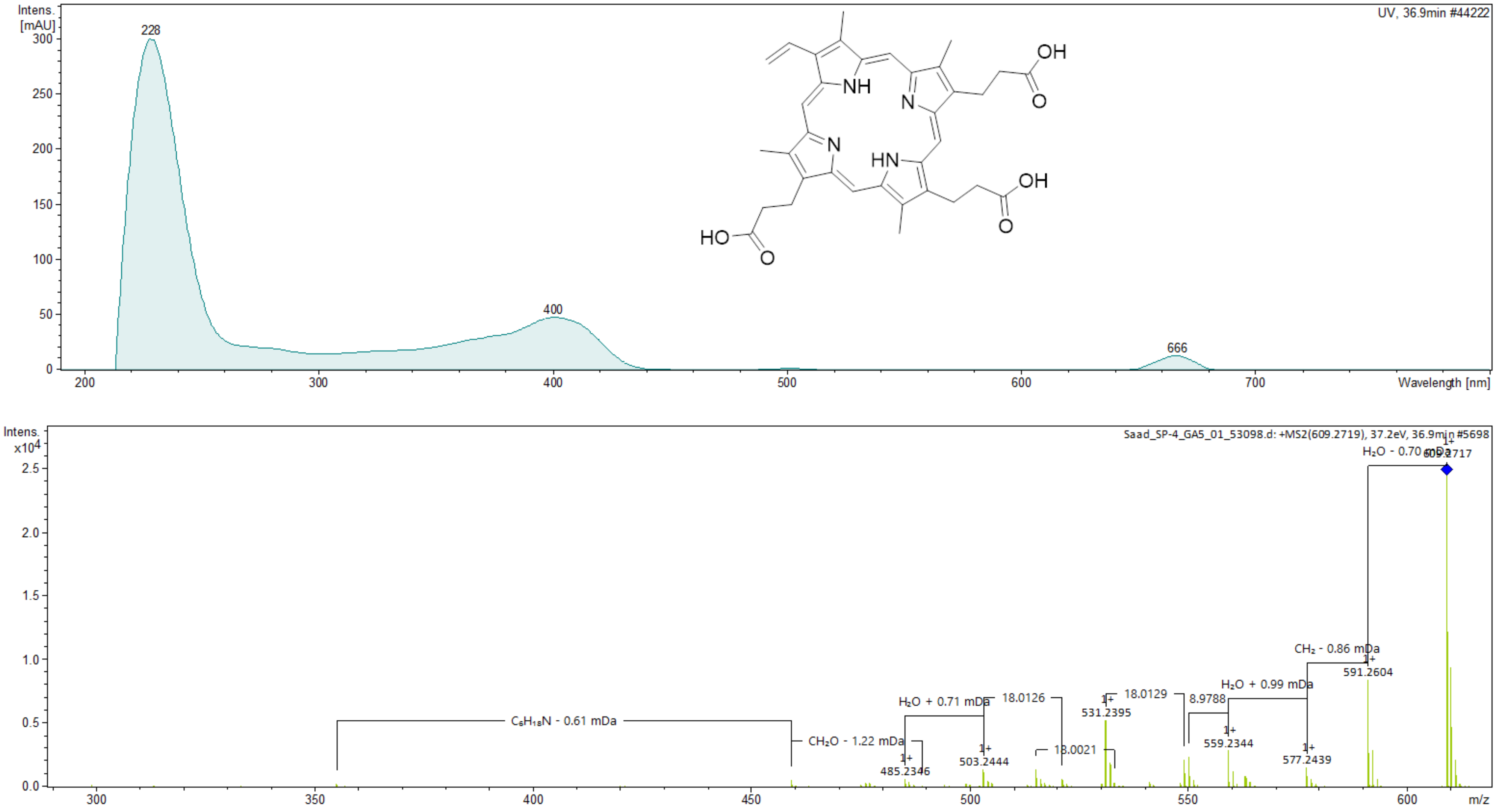

Fig. S16 OPLS score plot derived from GC/MS dataset modelling of Spirulina (S1, S3 & S4), Amphora (Am.), Chlorella (Chl.) samples against one another (A). The S-plot (B) shows the covariance p[1] against the correlation p(cor)[1] of the variables of the discriminating component of the OPLS-DA model. Cut-off values of P<0.05 were used; selected variables are highlighted in the S-plot with identifications are discussed in text**.**

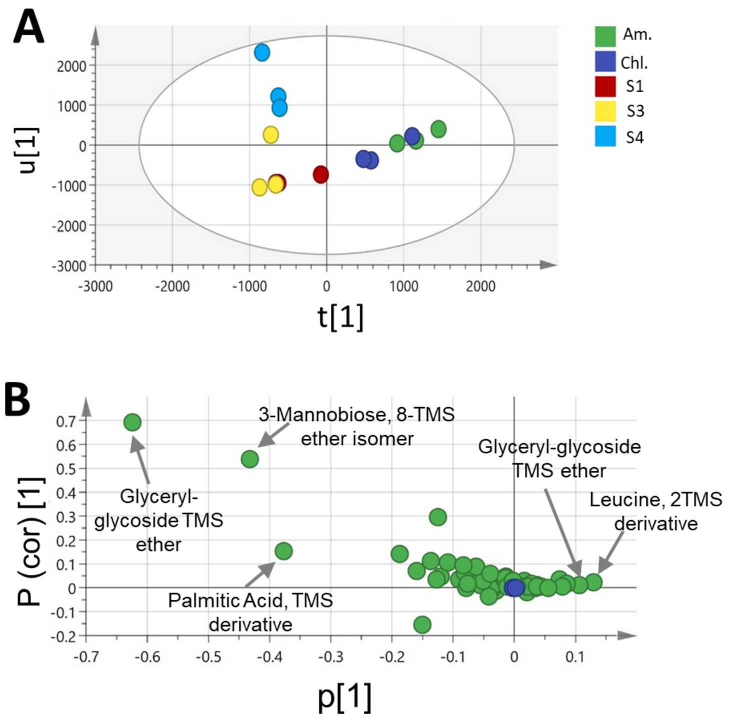

Fig. S17 OPLS-DA model optimization and validation parameters for modeling Spirulina (S3 & S4) samples against Amphora (Am.) samples based on GC/MS derived dataset. **A.** The diagnostic metrics R2Y and Q2 as function of number of principal components. **B**. Root mean square error of cross validation results using 7-fold cross validation method as function of number of principal components. **C.** Permutation test, n = 200 that showed negative Q2 intercept value. **D.** The receiver operating characteristic (ROC) curve to assess the classification ability of the developed OPLS-DA showing AUC = 1. **E.** CV-ANOVA to assess for model statistical significanc.

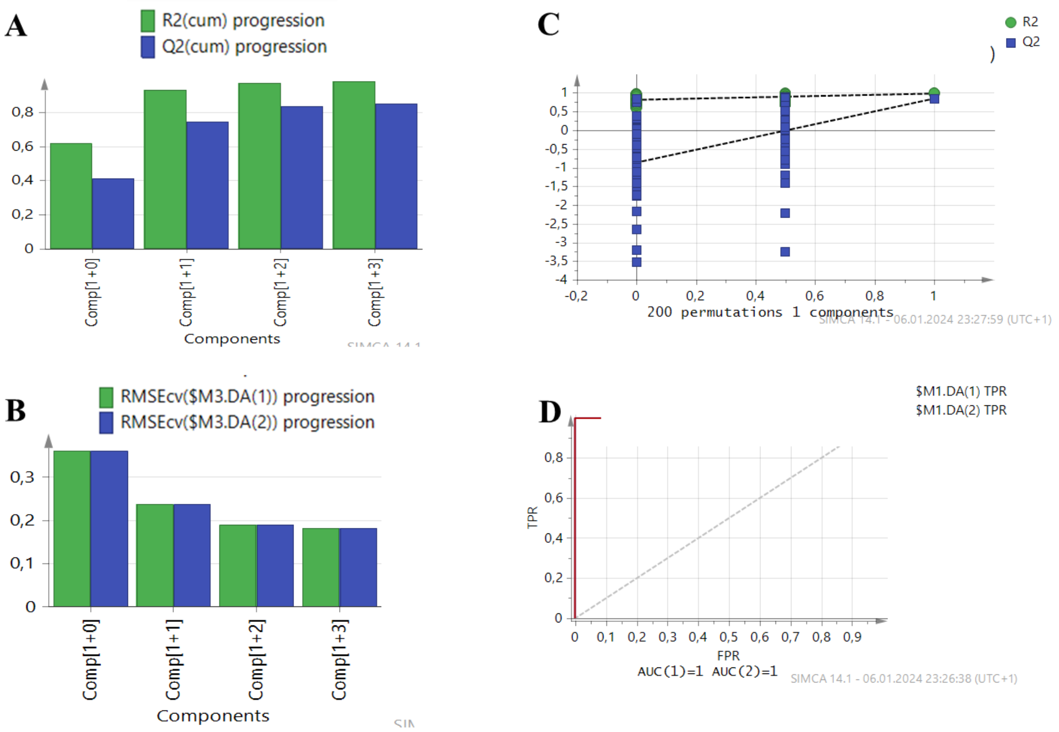

E

M1(Untitled)	SS	DF	MS	F	p	SD
Total corr.	8	8	1			1
Regression	6.80449	6	1.13408	1.89723	0.384659	1.06493
Residual	1.19551	2	0.597757			0.773147

Fig. S18 OPLS-DA model optimization and validation parameters for modeling Spirulina (S3 & S4) samples against Chlorella (Chl.) samples based on GC/MS derived dataset. **A.** The diagnostic metrics R2Y and Q2 as function of number of principal components. **B.** Root mean square error of cross validation results using 7-fold cross validation method as function of number of principal components. **C.** Permutation test, n = 200 that showed negative Q2 intercept value. **D.** The receiver operating characteristic (ROC) curve to assess the classification ability of the developed OPLS-DA showing AUC = 1. **E.** CV-ANOVA to assess for model statistical significanc.

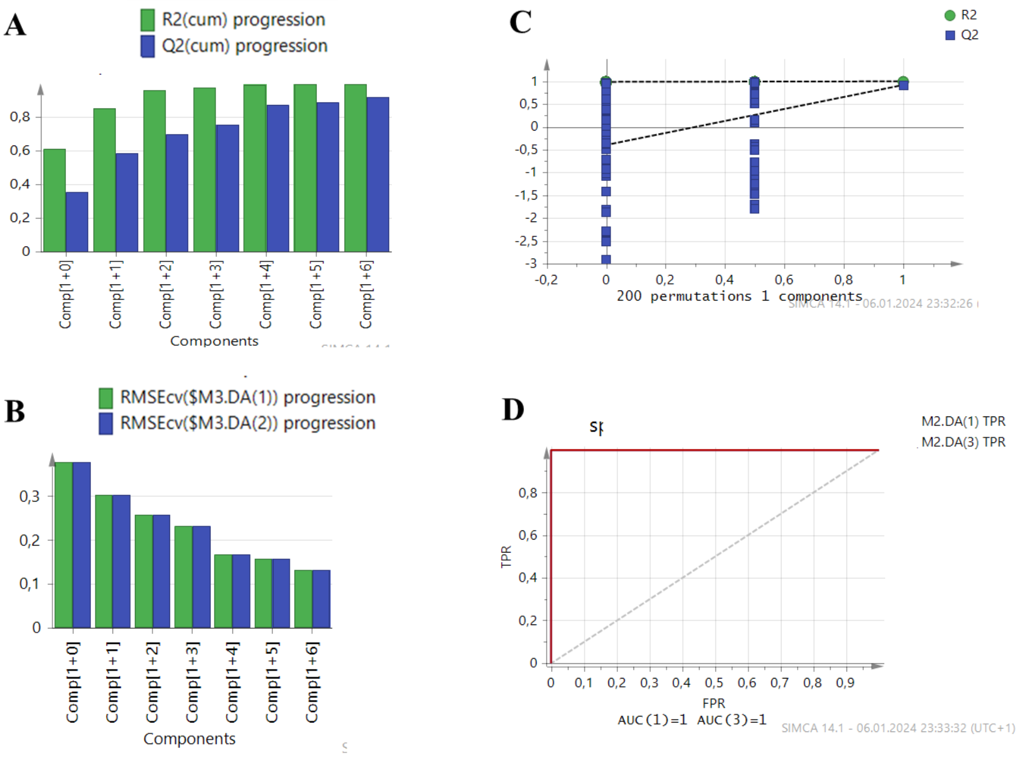

E

M2(Untitled)	SS	DF	MS	F	p	SD
Total corr.	8	8	1			1
Regression	7.3694	7	1.05277	1.66948	0.535705	1.02605
Residual	0.630599	1	0.630599			0.794103

Fig. S19 UV-Vis spectra of Spirulina, Amphora and Chlorella samples under study

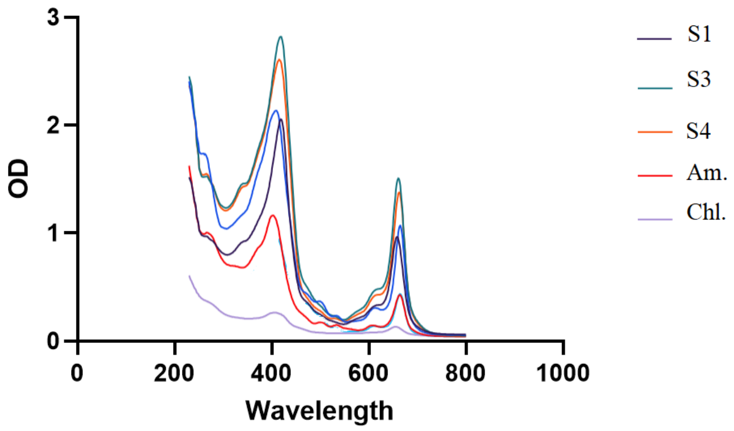

Fig. S20 Principal component analysis and hierarchical clustering of UV-Vis dataset of Spirulina (S1, S3 & S4), Amphora (Am.) and Chlorella (Chl.). (A) Score plot of PC1 vs. PC2 scores. (B) Loading plot for PC1 & PC2 contributing metabolites and their assignments. The metabolome clusters are located at the distinct positions in two-dimensional space described by two vectors of principal component 1 (PC1) = 98% and PC2 = 1.7%. (C) HCA plot.

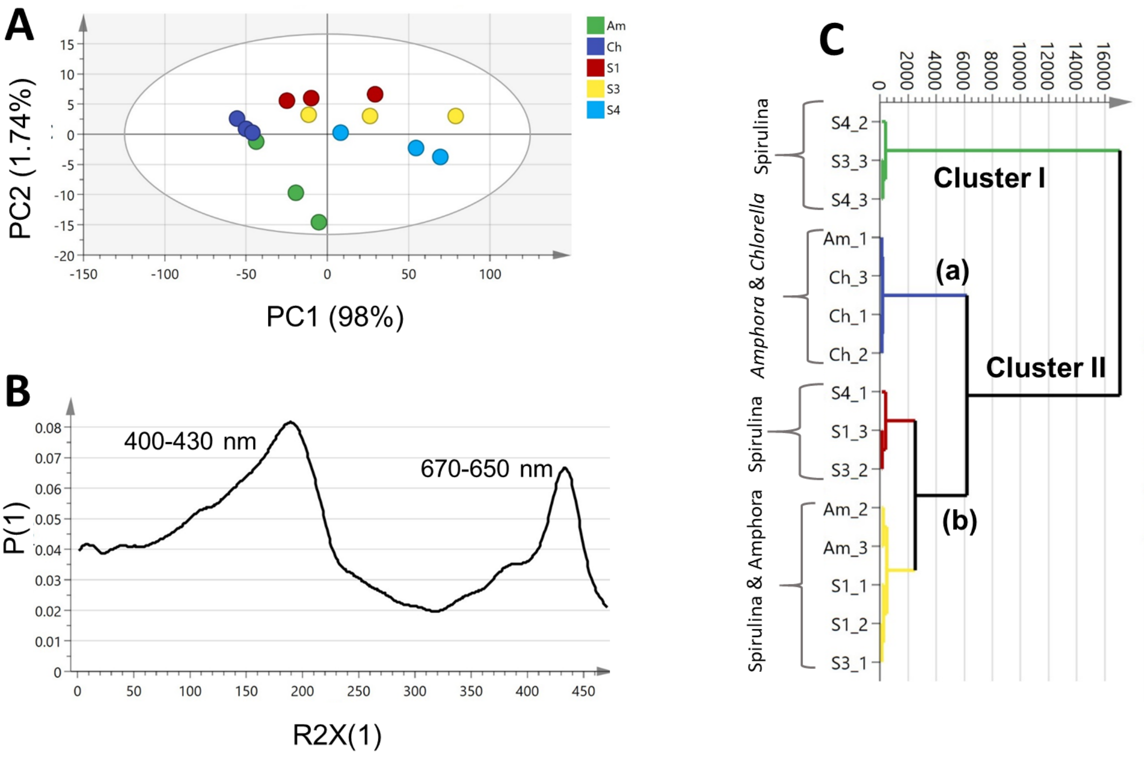

Fig. S21 OPLS-DA model optimization and validation parameters for modeling Spirulina (S3 & S4) samples against Amphora (Am.) samples based on UV-Vis derived dataset . **A.** The diagnostic metrics R2Y and Q2 as function of number of principal components. **B**. Root mean square error of cross validation results using 7-fold cross validation method as function of number of principal components. **C.** Permutation test, n = 200 that showed negative Q2 intercept value. **D.** The receiver operating characteristic (ROC) curve to assess the classification ability of the developed OPLS-DA showing AUC = 1. **E.** CV-ANOVA to assess for model statistical significanc.

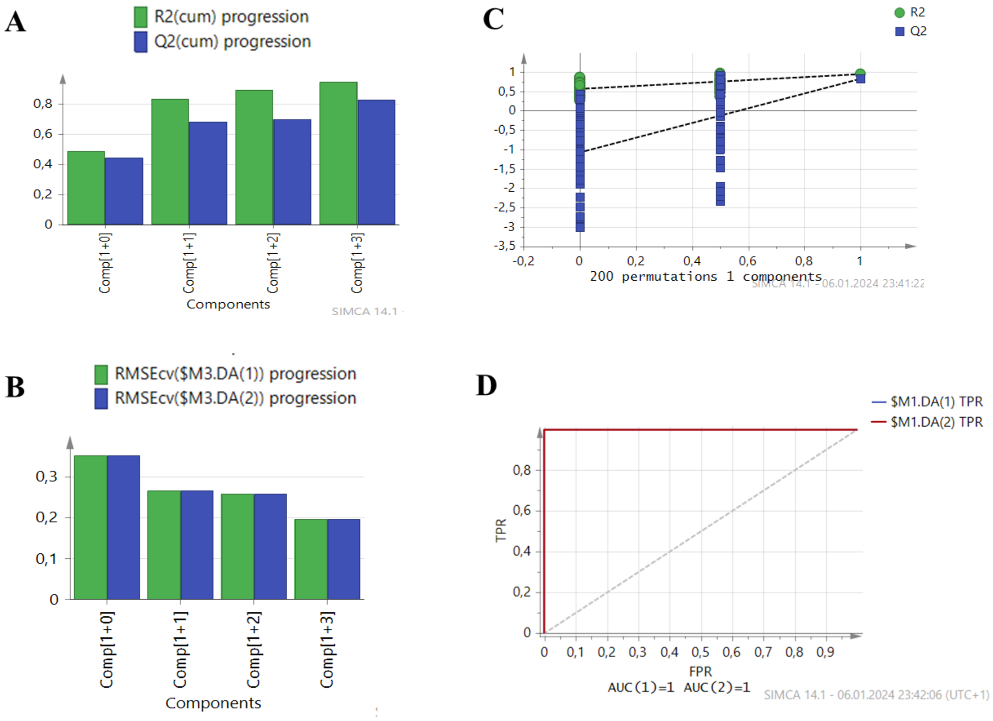

E

M1(Untitled)	SS	DF	MS	F	p	SD
Total corr.	8	8	1			1
Regression	6.60905	6	1.10151	1.58382	0.436172	1.04953
Residual	1.39095	2	0.695476			0.833952

Fig. S22 OPLS-DA model optimization and validation parameters for modeling Spirulina (S3 & S4) samples against Chlorella (Chl.) samples based on UV-Vis derived dataset. **A.** The diagnostic metrics R2Y and Q2 as function of number of principal components. **B.** Root mean square error of cross validation results using 7-fold cross validation method as function of number of principal components. **C.** Permutation test, n = 200 that showed negative Q2 intercept value. **D.** The receiver operating characteristic (ROC) curve to assess the classification ability of the developed OPLS-DA showing AUC = 1. **E.** CV-ANOVA to assess for model statistical significanc.

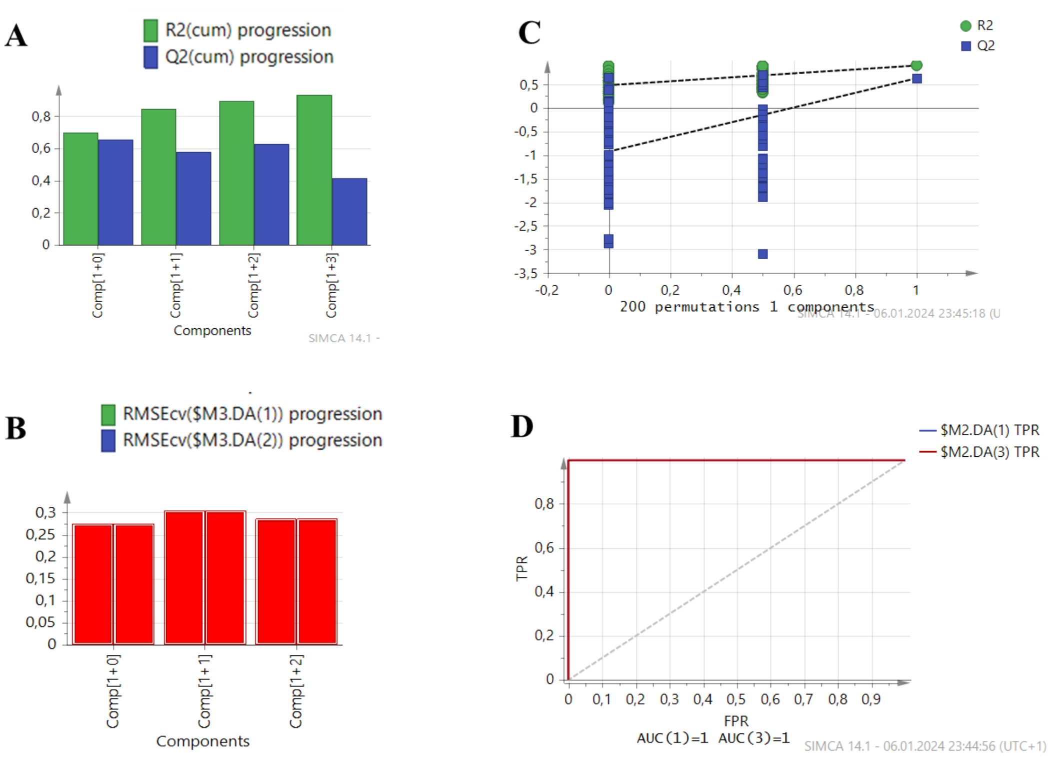

E

M2(Untitled)	SS	DF	MS	F	p	SD
Total corr.	8	8	1			1
Regression	5.03481	5	1.00696	1.01878	0.528425	1.00347
Residual	2.96519	3	0.988398			0.994182

References

1. Van Anh, N., et al., *Thladiantha seed oils-New source of conjugated fatty acids: characterization of triacylglycerols and fatty acids.* Journal of Oleo Science, 2020. **69**(9): p. 993-1000.
2. Liu, X.-T., et al., *Cholestane glycosides and trihydroxy fatty acids from the rhizomes of Dioscorea septemloba.* 2010. **76**(03): p. 291-294.
3. Kim, C.S., et al., *Five new oxylipins from Chaenomeles sinensis.* 2014. **49**(11): p. 1151-1159.
4. Hamburger, M., et al., *Plant anticancer agents, XLIII.(E, E)-7, 12-dioxo-octadeca-8, 10-dien-1-oic acid (ostopanic acid), a cytotoxic fatty acid from Ostodes paniculata.* 1987. **50**(2): p. 281-283.
5. DellaGreca, M., et al., *Isolation and Phytotoxicity of Apocarotenoids from Chenopodium a lbum.* 2004. **67**(9): p. 1492-1495.
6. Hegazi, N.M., et al., *Molecular networking leveraging the secondary metabolomes space of Halophila stipulaceae (Forsk.) Aschers. and Thalassia hemprichii (Ehrenb. ex Solms) Asch. in tandem with their chemosystematics and antidiabetic potentials.* 2021. **19**(5): p. 279.
7. Jin, Y., et al., *Comprehensive characterization of the chemical composition of Lurong dabu decoction and its absorbed prototypes and metabolites in rat plasma using UHPLC–Q Exactive Orbitrap–HRMS.* Food Research International, 2022. **161**: p. 111852.
8. Bertin, M.J., et al., *Identification of toxic fatty acid amides isolated from the harmful alga Prymnesium parvum carter.* Harmful algae, 2012. **20**: p. 111-116.
9. Ballesteros-Torres, J.M., et al., *Amino acids and acylcarnitine production by Chlorella vulgaris and Chlorella sorokiniana microalgae from wastewater culture.* PeerJ, 2019. **7**: p. e7977.
10. Vigor, C., et al., *Isoprostanoids quantitative profiling of marine red and brown macroalgae.* Food chemistry, 2018. **268**: p. 452-462.
11. Heiss, E.H., et al., *Identification of chromomoric acid CI as an Nrf2 activator in Chromolaena odorata.* Journal of natural products, 2014. **77**(3): p. 503-508.
12. Řezanka, T. and I.A. Guschina, *Glycoside esters from lichens of central Asia.* Phytochemistry, 2001. **58**(3): p. 509-516.
13. Luo, G., et al., *UHPLC-Q-Orbitrap-MS-Based Metabolomics Reveals Chemical Variations of Two Types of Rhizomes of Polygonatum sibiricum.* 2022. **27**(15): p. 4685.
14. Rho, M.-C., et al., *A novel monogalactosylacylglycerol with inhibitory effect on platelet aggregation from the cyanophyceae Oscillatoria rosea.* Journal of natural products, 1996. **59**(3): p. 308-309.
15. Qiu, S., et al., *Chemometrics-assisted identification of anti-inflammatory compounds from the green alga Klebsormidium flaccidum var. Zivo.* Molecules, 2020. **25**(5): p. 1048.
16. Le Bris, S., M.-R. Plante-Cuny, and E. Vacelet, *Characterisation of bacterial and algal pigments and breakdown products by HPLC in mixed freshwater planktonic populations.* Archiv für Hydrobiologie, 1998: p. 409-434.
17. Tang, Y., et al., *Comparative metabolomics analysis of Elodea nuttallii and Cladophora sp. in aquaculture systems.* Aquaculture, 2023. **563**: p. 738950.
18. Cheng, H.-H., et al., *Cytotoxic pheophorbide-related compounds from Clerodendrum c alamitosum and C. cyrtophyllum.* Journal of natural products, 2001. **64**(7): p. 915-919.
19. Sommella, E., et al., *Fast profiling of natural pigments in different spirulina (arthrospira platensis) dietary supplements by DI-FT-ICR and evaluation of their antioxidant potential by pre-column DPPH-UHPLC assay.* Molecules, 2018. **23**(5): p. 1152.
20. Li, J., et al., *Lipid metabolic characteristics and marker compounds of ripened Pu-erh tea during pile fermentation revealed by LC-MS-based lipidomics.* Food Chemistry, 2023. **404**: p. 134665.
21. Hirao, S., et al., *Algicidal activity of glycerolipids from brown alga Ishige sinicola toward red tide microalgae.* 2012: p. 1201312780-1201312780.
